# Disease profile and health-related quality of life (HRQoL) using the EuroQol (EQ-5D + C) questionnaire for chronic metallic mercury vapor intoxication

**DOI:** 10.1186/s12955-015-0388-0

**Published:** 2015-12-09

**Authors:** Nadine Steckling, Dietrich Plass, Stephan Bose-O’Reilly, Alfred Bogomir Kobal, Alexander Krämer, Claudia Hornberg

**Affiliations:** WHO Collaborating Centre for Occupational Health, Institute and Outpatient Clinic for Occupational, Social and Environmental Medicine, Workgroup Paediatric Environmental Epidemiology, University Hospital Munich, Ziemssenstr. 1, D-80336 Munich, Germany; School of Public Health, Department of Environment & Health, Bielefeld University, Universitätsstraße, 25, D-33615 Bielefeld, Germany; Federal Environment Agency, Section Exposure Assessment and Environmental Health Indicators, Corrensplatz 1, D-14195 Berlin, Germany; Institute of Public Health, Medical Decision Making and Health Technology Assessment, Department of Public Health and Health Technology Assessment, Eduard Wallnoefer Center I, UMIT (The Health & Life Sciences University), A-6060 Hall i.T., Austria; Department of Occupational Medicine, Idrija Mercury Mine, Idrija, Slovenia; School of Public Health, Department of Public Health Medicine, Bielefeld University, Universitätsstraße 25, D-33615 Bielefeld, Germany

**Keywords:** Mercury intoxication, Health-related quality of life (HRQoL), EuroQol (EQ-5D + C-3L), Expert elicitation, Disability weight

## Abstract

**Background:**

Toxic mercury is still being used today for example by workers mining gold, resulting in diverse health symptoms in users and individuals in proximity. A considerable burden of disease (BoD) can be assumed, while previous analyses were limited by data scarcity. Besides limited epidemiological data, neither data about the health-related quality of life (HRQoL) nor about the disease severity (disability weight, DW) is available. The aim of the project was to develop disease profiles of chronic metallic mercury vapor intoxication (CMMVI) by including the HRQoL to improve the data basis for BoD analyses of gold miners exposed to mercury.

**Methods:**

Disease profiles comprising the disease label [a], differentiation into disease stages [b], description of the cause of exposure [c], a list of common symptoms [d], and an assessment of the HRQoL [e] were developed using expert elicitation and literature search. The HRQoL was assessed by experts using the five EuroQol dimensions accompanied by the cognition add-on questionnaire (EQ-5D + C).

**Results:**

The ten sources used for the analyses (interview transcript, presentation, and eight literature reviews) identified more than 250 terms describing 85 distinguishable health effects of CMMVI. The analysis revealed 29 common symptoms that were frequently mentioned. Moderate and severe CMMVI cases differ regarding their symptoms and/or symptom severity and HRQoL, resulting in the EQ-5D + C-3L codes 121222 and 233333, respectively.

**Conclusions:**

The profiles should be used to facilitate the ascertainment of CMMVI cases, to compare the HRQoL with other diseases, to derive DWs for improving BoD estimates, and to foster discussions about how to reduce the associated burden.

**Electronic supplementary material:**

The online version of this article (doi:10.1186/s12955-015-0388-0) contains supplementary material, which is available to authorized users.

## Background

The use of mercury in artisanal small-scale gold mining (ASGM) is a problem of global concern [1] because it leads to the greatest anthropogenic emission of this toxic heavy metal worldwide [2, 3]. Mercury is extensively used in ASGM to extract gold, because amalgamation does not require high technologies [4]. Mercury binds the gold of the crushed ore. The resulting amalgam is smelted so that the mercury vaporizes and the gold remains [5]. This procedure exposes and endangers not only the miners and but also residents in close proximity to enormous quantities of mercury [6].

More than 16 million miners worldwide, especially in Africa, Asia, and South America, are assumed to use mercury for gold extraction, while the mercury release is highest in countries with the lowest technology [4]. Most of these workers belong to the poorest segments of the global society [7] and live outside the regulating systems with respect to prevention, reporting, and compensation of occupational diseases [8]. As a consequence, their diseases often are undetected and -reported and the associated burden is ignored or not recognized [8]. A considerable burden of disease (BoD) can be assumed, although previous BoD analyses were limited by data scarcity [9–13].

The BoD concept allows estimating and comparing the health impact of (risk factor attributable) diseases expressed as disability-adjusted life years (DALYs) by combining mortality and morbidity into a single metric [14–16], while the morbidity is associated with the health-related quality of life (HRQoL) [17]. The mortality part of the DALY – expressed as years of life lost due to premature death (YLL) – is calculated by multiplying the number of deaths with a standard remaining life expectancy at the age of death. The morbidity part is determined by the years lived with disability (YLD) while prevalence cases (or alternative incident cases multiplied with duration of the disease) are multiplied with a disability weight (DW) [15, 18, 19].

The DW is a factor between one, what means a health state comparable with death, and zero, a health state without disability [15]. In several studies, panels of experts or other subgroups were asked to assess the severity of disease states (e.g., [15, 20–22]). Although more than 200 disease stages were determined in the largest study [22], specific conditions like the chronic intoxication due to mercury exposure in ASGM were not considered. A missing DW is a common limitation when considering underexplored health effects. For this reason, in previous studies either provisional DWs were used [9, 11] or the analyses were not carried out [10]. However, determining the BoD of health effects with limited data is especially important to ensure the awareness of policy makers and to avoid an implicit assumption, that these causes have no burden [23].

Although there are several ways to derive DWs, they all heavily rely on descriptions of health states which can be evaluated by different respondent groups. The descriptions used vary widely, but can generally be divided into three major sub-groups: disease-specific descriptions, generic descriptions, and descriptions combining the aforementioned approaches. Using information about the HRQoL as generic description and combining it with disease-specific information has been done previously [20, 21, 24–28] and is recommended by Haagsma and colleagues who recently reviewed and compared DW studies [29].

The aim of the DiWIntox-1 project (first part of the project ‘disability weights for chronic mercury intoxication’) was to develop disease profiles for chronic intoxications due to mercury in ASGM comprising disease-specific and generic components by including information about the HRQoL. The descriptions should be useful as a starting point to derive DWs for the health outcome of interest. The paper focusses on the following research questions: [a] How can the health outcome of mercury exposed ASGM workers be labelled? [b] In what way can the health outcome of interest be differentiated in disease stages? [c] What is the characteristic exposure situation of ASGM workers using mercury? [d] Which health symptoms are common for the health outcome of interest? [e] What is the HRQoL of individuals showing the health outcome of interest?

## Methods

Based on a similar procedure used in previous studies [20, 21, 30], the following components were included in the establishment of disease profiles for the DWs: a disease label [a], classification into disease stages [b], the exposure setting [c], a list of common symptoms [d], and an assessment of the HRQoL [e]. For differentiation into disease stages [b], information on symptom severity and probability of occurrence (common or uncommon, degree of severity) were needed. The information about the exposure [c] and symptoms [d] form the disease-specific part of the description. The assessment of the HRQoL [e] belongs to the generic part of the description. The information for the disease profiles (components a to e) was obtained by consulting experts and conducting a systematic literature review. Finally, the information from the different sources was combined.

### Expert elicitation

An expert meeting in Ljubljana, Slovenia, was conducted in December, 2012. The meeting agenda consisted of oral presentations and a group interview (Additional file 1; File S1). One of the presentations (*Chronic Hg Intoxication in the Idrija Mercury Mine;* Alfred Kobal) and the interview were used as data sources for the disease profiles. A list of references included in the presentation is given in Additional file 1: File S2.

The methodological design of the interview was based on a formal expert elicitation protocol developed by Knol et al. [31] and Slottje et al. [32]. The goal was to determine and describe all components of the disease profiles listed above (components [a] to [e]). The interview was recorded and transcribed.

The discussion group was formed by four Slovenian and one German expert, one interviewer and one student assistant (both German). The experts were trained in medicine, toxicology, or public health, and were experienced with the effects of mercury on human health (Additional file 1; File S3). The two medical doctors had direct experiences with medical examinations of workers chronically exposed to mercury, either workers from Idrija mercury mine in Slovenia, which was closed in 1995, or with ASGM workers and residents at mining sites in several countries.

The interview guideline comprised 27 questions; 24 were used to determine the exposure and health outcome of interest, and three for the evaluation of the meeting (Additional file 1; File S4). The questions were designed to guide the discussion in order to meet the objectives of the project. In addition to the consensus discussions about disease-specific characteristics, a generic approach was used to assess the HRQoL applying the five EuroQol dimensions (mobility, self-care, usual activities, pain/discomfort, anxiety/depression) plus a cognition questionnaire (EQ-5D + C) [33–35] (Additional file 1; File S4; questions 19–24). In the group interview, questions were answered in an open discussion. Neither structured instruments (e.g., the Delphi method) nor consecutive rounds of written expert opinions were employed.

The EuroQol group was informed about the EQ-5D + C application and confirmed its consent. The original protocol for EQ-5D questionnaires [34] was adapted, with experts rather than patients being interviewed (*Please indicate which statement best describes the health state of a person with the disease*). In a consensus discussion, the participants assessed symptom severity according to the six dimensions coded in three levels (EQ-5D + C-3L), defined as 1 for no problems, 2 for problems, and 3 for severe problems.

### Systematic literature review

In addition to the interview, a literature review was conducted after the meeting to accurately focus on the health outcome and exposure setting determined during the interview. The aims were to contribute to the components [b] and [d]; namely to complete the list of symptoms and collect information about symptom severity and the probability of occurrence at different stages of the disease.

**Fig. 1:**
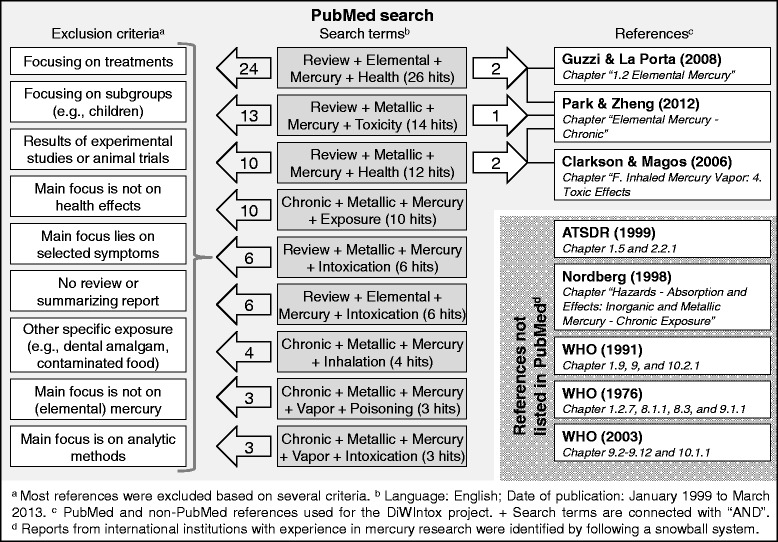
Flowchart of the literature search and references used

Two approaches ensuring a comprehensive overview of the published literature by identifying references listed in PubMed and references not listed in PubMed (see Fig. 1) were used. First, reports from international institutions with experience in mercury research were obtained using a snowball system. Reports from the World Health Organization (WHO) [36–38], the Agency for Toxic Substances and Disease Registry (ATSDR) [39] and the Encyclopaedia of Occupational Health & Safety from the International Labour Organization [40] were identified as important sources to find information about chronic health effects of mercury exposure in gold mining (see bottom right corner in Fig. 1). Reports from other international institutions about mercury were excluded because of other main foci (e.g., food (EFSA), environment (UNEP), industrial development (UNIDO)). Second, a PubMed search was done to find reviews on the predefined exposure and health outcome of interest. The keywords, *review, elemental/metallic, mercury, exposure/health/intoxication/poisoning/toxicity, chronic,* and *vapor* were combined to find sufficient search terms. Criteria for exclusion, like a focus on treatments or experimental studies, were applied which are listed in Fig. 1. The PubMed search was restricted to articles published between 1999 and March 2013 because a comprehensive report reviewing relevant literature [39] was published in 1999. All references included were screened to identify relevant chapters about symptoms, caused by the predefined exposure in humans, and their severity and probability of occurrence.

When screening the literature, difficulties were faced in identifying symptoms specifically attributable to the exposure of interest. In some cases, information about the form of mercury and the duration or pathway of exposure was missing in the references used. Only those symptoms were included where information on the exposure was available.

### Combining information from different sources

In total, the disease profiles are based on ten sources belonging to three types of sources: The transcript of the interview, one presentation from the expert meeting, and eight literature references [36–43] (Fig. 1). The information about common symptoms, probability of symptom occurrence, and symptom severity was extracted from the interview transcript and presentation slides and complemented by the information identified in the literature (Additional file 1; File S5).

The list of symptoms was structured by applying categories of the system or part of the body affected as used in the considered literature while some adaptions were necessary (Additional file 1; File S5; column A). The advanced search-function of the online ICD-10 list [44] was used to ensure that symptoms were assigned to the correct categories and to identify synonyms describing the same symptom.

The condensed disease profiles were prepared on the basis of the comprehensive list of symptoms (Additional file 1; File S5). Symptoms were included in the disease-specific description of the profiles if they were confirmed by more than two sources, and – if available – described as a common symptom, or at least not as uncommon. Consequently, the profiles do not contain a complete list of symptoms but the common and frequent mentioned. Where several terms were used for the same symptom (Additional file 1; File S5; column B), one term was chosen for further usage (Additional file 1; File S5; column C). Information about symptom severity allowed the distinction in disease stages. Where no information about increasing or decreasing severity was available, the common symptoms were attributed to all stages (Additional file 1; File S5; columns D, E, and F).

The generic disease description is completely based on the interview because it was not possible to extract such specific information from the literature. The EQ-5D + C valuation was checked again by the experts of the initial discussion group after seeing the finalized disease-specific description to ensure suitability to the condensed list of symptoms. The final disease profiles were also checked by the experts.

## Results

The interview participants agreed to label the disease *chronic metallic mercury vapor intoxication* (CMMVI) to describe the health outcome of interest within the project [a]. The experts confirmed mild, moderate, and severe stages of CMMVI. However, for the project purpose, a dichotomous differentiation of stages (moderate and severe) seemed feasible. This became clear during the assessment of the HRQoL and during literature search. A distinction of CMMVI in three stages was less expedient while a sharp distinction was achieved when focusing on two disease stages [b]. The disease-specific description including the exposure [c] and health situation [d] and the generic description indicating the HRQoL of CMMVI [e] are described in detail below. The disease profiles of moderate and severe cases of CMMVI including the components [a] to [e] are presented in Table 1.

**Table 1:** Disease profiles of the moderate and severe cases of chronic metallic mercury vapor intoxication (CMMVI)

Disease label [a]	Chronic Metallic Mercury Vapor Intoxication (CMMVI)	
Disease stage [b]	Moderate case	Severe case
Disease-specific description (including exposure [c] and health situation [d])	Adults with high mercury body burden caused by chronic inhalation of metallic mercury vapor who show several of the following symptoms:	Adults with a very high mercury body burden caused by chronic inhalation of metallic mercury vapor who show several of the following symptoms:
	• Slight tremor of fingers, hands, and limbs; coordination problems; dysfunction of movement control; weakness • Reflexes abnormalities; peripheral nerve abnormalities; sensory disturbances • Sleep disorders; irritability; nervousness; fatigue; memory impairment; difficulty in concentration; shyness; depressive mood; loss of confidence; lack of self-control • Renal effects like enzymuria, proteinuria, and glomerular dysfunction, increased urinary excretion of N-acetyl-β-glucosaminidase (NAG) • Loss of appetite; salivation • Immunological changes	• Pronounced tremor in several parts of the body; severe coordination problems; dysfunction of movement control; weakness • Polyneuropathy • Insomnia; hyperirritability; nervousness; fatigue; loss of memory; difficulty in concentration; extreme shyness; depression; loss of confidence; lack of self-control; social avoidance • Abnormal renal function with enzymuria, high proteinuria, glomerular dysfunction, and rising urinary excretion of N-acetyl-β-glucosaminidase (NAG) • Anorexia; excessive salivation; gingivitis; stomatitis • Immunological changes • Difficulty seeing
Generic description (EQ-5D + C [e])	• No problems in walking (1) • Some problems with self-care (2) • No problems with performing usual activities (1) • Moderate pain or discomfort (2) • Moderately anxious or depressed (2) • Some problems in cognitive functions (2)	• Some problems in walking about (2) • Not able to wash or dress themselves (3) • Not able to perform usual activities (3) • Severe pain or discomfort (3) • Extremely anxious or depressed (3) • Severe problems in cognitive functions (3)

[a] to [e]: A description of the components [a] to [e] is given in the chapter “Methods”. (1), (2), (3): EQ-5D + C code; (1) no problems, (2) problems, (3) severe problems. EQ-5D + C: the five EuroQol dimensions (mobility, self-care, usual activities, pain/discomfort, anxiety/depression) plus cognition (EQ-5D + C) questionnaire

### Disease-specific description

The exposure of the case of interest is described as adults chronically exposed to metallic mercury vapors, corresponding to the main exposure of ASGM workers and mercury miners [c]. Hence, individuals similarly exposed to mercury, e.g., due to accidents [45] or by working in other industries with comparable exposure settings like in industries which use or emit mercury, such as power and heating plants (due to fossil fuel combustion), metal and cement production, waste incineration, or mercury mining [3], also fall under the general description of the exposure setting. Consequently, the disease profiles developed can be used if the exposure scenario (form of mercury, exposure pathway, etc.) is the same as in ASGM. All other types of mercury exposure (e.g., other forms of mercury, exposure pathways, or durations of exposure or dosages), other concomitant exposures (e.g., other chemicals, other hazards such as noise or accidents), or comorbidities (e.g., acute intoxications, cancer, silicosis, cardiovascular or infectious diseases) were not considered for the current definition of CMMVI. Examples for other mercury-related health outcomes are Minamata disease, acrodynia/pink disease, mild mental retardation, kidney damage, or acute intoxications, which might have similar but also different clinical symptoms [39] than CMMVI. Due to the focus on adult gold miners, children were excluded from the case description.

The complete list of symptoms as identified in the ten sources used is included in Additional file 1; File S5. More than 250 synonymous terms of symptoms (including nearly 60 health signs like abnormal laboratory findings such as proteinuria) were summarized in 85 distinguishable symptoms and, with reference to ICD-10 and the categories used in the literature, assigned to dermal, endocrine, digestive, general, hematological, immunological and lymphoreticular, metabolic, neurological, renal, and respiratory effects. The neurological effects were further subsumed in categories depending on whether they affected the musculoskeletal system, led to behavioral, cognitive, emotional, or mental effects, or affecting other systems (auricular, ocular, speech).

**Fig. 2:**
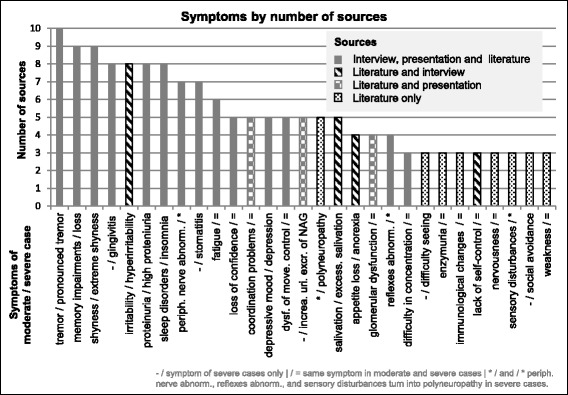
Visualization of the number of sources describing each symptom included in the disease profiles

Twenty-nine of the 85 symptoms were mentioned in more than two of the ten sources and included in the moderate and/or severe disease profiles ([d], Fig. 2). Considering the information about symptom severity and probability of occurrence, a list of 23 and 26 symptoms [d] were included in the disease profiles of moderate and severe cases, respectively (Table 1).

The symptoms included in the disease profiles are categorized as neurological effects regarding the musculoskeletal system (e.g., *tremor*) and in terms of behavioral, cognitive, emotional, and mental effects (e.g., *fatigue*, *loss of confidence*). For moderate CMMVI cases, the neurological symptoms *reflex abnormalities*, *peripheral nerve abnormalities*, and *sensory disturbances* are listed separately. For severe cases, the additional term *polyneuropathy*, meaning the presence of symptoms of the peripheral nerve system, was included. The neurological symptom *difficulty seeing* (effects on the ocular system) was included into the profile of the severe but not the moderate case. Furthermore, renal (e.g., *proteinuria*), digestive (e.g., *loss of appetite*), and immunological and lymphoreticular effects (*immunological changes*) were included in both profiles with different symptoms and/or symptom severities. By implication, dermal, endocrine, hematological, metabolic, respiratory effects, and neurological effects of the auricular system or speech, while reported symptoms of CMMVI, are identified as uncommon and/or rarely described and so were not included in the disease profiles.

For some symptoms, there are indications that in the most severe cases, new symptoms may occur either together with or instead of a symptom of a moderate case. Following the interview, the symptom *dry mouth* (effect of the digestive system) might replace *salivation* in very severe cases (Additional file 1;File S5 row 9). Moreover, *muscular spasms* (neurological effect of the musculoskeletal system) may occur simultaneously with extremely severe *tremor*, which is described in one literature reference [37] (Additional file 1; File S5; row 49). However, these symptoms were not included in the profiles, because they are mentioned in just one or two sources.

In most cases, symptoms rather than specific signs were included in the profiles (e.g., *tremor* was included while a *change in handwriting* was not explicitly mentioned). Exceptions are made regarding renal effects (Additional file 1; rows 75–89). The laboratory signs frequently mentioned, *enzymuria*, *proteinuria*, and *increased urinary excretion of N-acetyl-β-glucosaminidase (NAG),* were classified as *renal effects* (moderate case) and as *abnormal renal function* (severe case) and included in the profiles partly by including information about severity (e.g., *proteinuria* in the moderate case and *high proteinuria* in the severe case). The *nephrotic syndrome* is the only frequently mentioned symptom which was not included in the disease profile,s because it is described as uncommon [37] or rare [43] (Additional file 1; File S5; row 77).

*Peripheral nerve abnormalities* are mentioned in seven of ten sources (Additional file 1; File S5; row 33). One source describes it as prominent [38] but another as uncommon [42] while the remaining five sources give no information about the frequency of occurrence. This contradiction was resolved by treating *peripheral nerve abnormalities* as a common symptom because it is mentioned so often.

No reference reported an increase or decrease of the common CMMVI symptoms *loss of confidence*, *difficulty in concentration*, *dysfunction of movement control*, *enzymuria*, *fatigue*, *glomerular dysfunction*, *immunological changes*, *lack of self-control*, *nervousness*, and *weakness*. Consequently, they seemed to be independent from the severity level, which is why they were included in both disease profiles (moderate and severe case) without variation. All other symptoms included in the disease profiles were either described with increasing intensity (e.g., *irritability* and *slight tremor* in the moderate, and *hyperirritability* and *pronounced tremor* in severe cases) or with initial occurrence in severe cases (e.g., *difficulty seeing*).

**Fig. 3:**
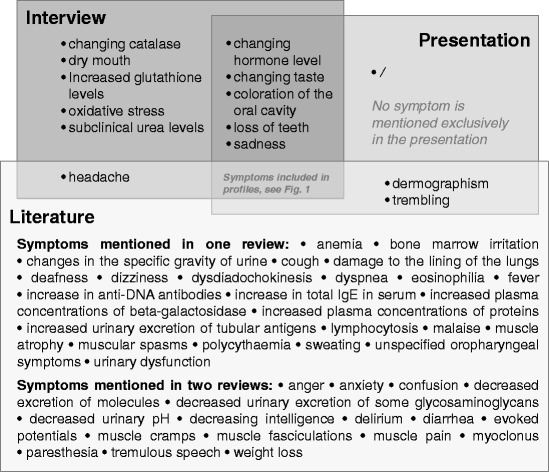
Symptoms not included in the disease profiles of CMMVI which were mentioned in only one or two sources

From the 29 symptoms included in the moderate and/or severe disease profiles, 14 are mentioned in the interview, the presentation, and the literature (Fig. 2). Because symptoms were included in the disease profiles if they appeared in more than two of the ten sources, symptoms mentioned only in the interview and/or the presentation but not in the literature were excluded. This was the case for *changing hormone levels* (endocrine effect), *changing taste*, *coloration of the oral cavity*, *dry mouth*, *loss of teeth* (all effects of the digestive system), *higher level of glutathione and changing catalase* (hematological effects), *oxidative stress* (metabolic effect), *sadness* (neurological effects in terms of behavioral, cognitive, emotional, mental effects), and *subclinical urea level*. Many symptoms also were excluded because they were mentioned in only one or two references (Fig. 3).

*Erethism*, frequently described as resulting from chronic exposure to metallic mercury vapors [36, 37, 39, 41–43] (Additional file 1; File S5; row 70), was not included in the profiles because it overlaps with other symptoms and its description changed over time [43]. In the profiles, the symptoms subsumed under *erethism* (e.g., irritability, shyness, loss of memory, etc.) were included.

### Generic description

The generic descriptions of moderate and severe cases, as determined by the expert interview following the EQ-5D + C-3L evaluation, were compared to a healthy person (Table 2) and included in the health profiles (Table 1). The expert elicitation yielded the assessment that the quality of life is effected regarding *mobility* and *usual activities* in severe but not in moderate cases. However, the criteria *self-care*, *pain*, *anxiety*, and *cognition* are always affected by CMMVI. However, their expression (problems or severe problems) depends on the severity of the disease.

**Table 2:** Visualization of the restrictions in the health-related quality of life (HRQoL) when comparing moderate and severe cases with healthy person

Criteria	Healthy persons	Moderate CMMVI cases	Severe CMMVI cases
Mobility	1	1	2
Self-care	1	2	3
Usual activities	1	1	3
Pain/discomfort	1	2	3
Anxiety/depression	11	2	3
Cognition		2	3
EQ-5D + C-3L Code	111111	121222	233333

1: No restrictions in the health-related quality of life (HRQOL)

2: Moderate restrictions in the HRQoL

3: Severe restrictions in the HRQoL

In moderate cases, some problems with self-care and cognitive functions were assumed to be accompanied by moderate pain or discomfort in a moderately anxious or depressed individual. Problems in walking about or performing usual activities were not assumed. On the other hand, restrictions in all six categories (problems or even severe problems) are expected in severe cases. Individuals with a severe CMMVI may have some problems with walking about, but severe problems with cognitive functions, severe pain or discomfort, be extremely anxious or depressed, not able to wash or dress themselves or to perform usual activities. Following the EQ-5D + C coding system, the moderate and severe cases were valued as 121222 and 233333, respectively.

## Discussion

Disease profiles of moderate and severe CMMVI were developed according to the specific exposure situation of ASGM, including a list of common symptoms in the disease-specific description and EQ-5D + C-3L codes in the generic description. Information from the literature (8 selected reviews) was combined with expert knowledge (interview transcript and presentation) to address the research questions. This is the first time that a mercury-related health outcome was assessed with EQ-5D + C and, generally, regarding its HRQoL.

A total of 85 distinct symptoms were extracted from the literature and the expert interview. This broad range of health effects was narrowed down to the essential information to distinguish two severity levels of CMMVI, including only frequently mentioned symptoms in the disease specific descriptions. All symptoms included in the description of moderate CMMVI cases are represented in the description of the severe cases, but more than half of the symptoms worsen from moderate to severe cases. A few symptoms were assumed to occur only in severe cases. The descriptions of symptoms extracted from the ten sources used are very unspecific in some cases (e.g., immunological changes; difficulty seeing).

The aim of the project was to develop disease profiles for the derivation of DWs to improve the data basis for estimating the BoD due to CMMVI as result of the mercury use in ASGM. Several methodological choices are possible for DW derivation; however, in addition to the description of health states, the valuation method and the composition of the panel are key components. Pairwise comparisons, time or person trade-off (TTO, PTO) and visual analogue scale (VAS) are established methods for assessing the severity of health states. A panel consisting of mostly experts or other subgroups such as samples of the general population or patients is asked to assess the severity of different health states. The health states were presented as disease-specific and/or generic descriptions [29]. The assessments of the panel are then converted to the DW, which ranges between zero and one [22, 29]. The disease profiles of CMMVI are designed for use in such DW derivations. The profiles are developed in a means comparable to profiles of other diseases already published. Haagsma and colleagues [29] identified six DW studies using both disease-specific and the generic EQ-5D description for disease profiles, also [20, 21, 25–28]. However, the decision which available set of disease profiles are used for comparisons with the profile of CMMVI might be followed by further adaptions of the disease-specific part. The reasons are individual differences of the sets of profiles like the length of descriptions or writing styles.

Further, distinctions in more than two disease stages seemed to be possible because some single symptoms may worsen before others (Additional file 1; File S5; columns D and E). For example, neurological and renal effects are generally seen earlier than effects in other organs [38]. Hence, slight renal involvement might appear before neurological effects, while chronologically the central nervous system was assumed to be affected before the kidneys [37, 40]. Furthermore, *tremors* occur a little after minor psychological disturbances (e.g., *shyness*) [36] while psychological, renal, and neurological effects can be assumed before other organs are involved. Although further distinctions seemed possible, very detailed and individual disease profiles are not useful from a public health point of view, when aiming on improving the data basis for BoD analyses. However, it must be kept in mind that health and disease are distributed differently amongst individuals and are influenced by factors like the environment, lifestyle, and genetics (exposome) [46]. There is individual variation in mercury intoxication [36, 40, 43] while several symptoms are unspecific and do not always appear [43] and even subclinical intoxications are possible [47].

The unspecificity of the symptoms became clear when using the ICD-10 list to arrange the symptoms in categories. Most symptoms are listed in the ICD-10 chapter R00-R99, *Symptoms, signs and abnormal clinical and laboratory findings, not elsewhere classified*. Due to the generality of the symptoms, their occurrence is not limited to chronic exposure to elemental mercury vapors but to a number of other diseases and causes.

The disease profiles are developed for a strictly defined case description. Specific topics were excluded, although they are relevant to the use of mercury in gold mining and should be subject of future research. The profiles focus on adults while children were excluded. The specific health outcomes of children exposed to mercury in ASGM should definitely be included in future research because child labor is very common in gold mining [7]. Separate disease profiles should be developed with a focus on children because it is likely that they are affected differently than adults. For example, children might develop a skin reaction called acrodynia what is not common in adults.

A further subject of future research should be comorbidities. Although comorbidities are very common in the occupational settings described [48–50], they should not be addressed until single risk hazards have been identified. For example, the Global Burden of Disease 2010 Study considers co-morbidities when deriving DWs and not earlier in the disease descriptions [22].

The disease profiles should be incorporated into diagnostic criteria for epidemiological data surveys. Some surveys about mercury exposure of gold miners are available and their data should be examined to identify cases of CMMVI. Some existing surveys use a diagnostic tool developed by Drasch et al. (2001) [51] to identify chronic mercury intoxications [51–54]. The tool combines information on the concentration of mercury in human biomonitors and health effects, while no distinction was made regarding the form of mercury and symptoms. This diagnostic tool should be compared with the disease profiles in this paper and can be used as starting point to define criteria for diagnosing CMMVI.

The project was based on published information and expert elicitation, while consulting the affected individuals themselves was not an option. Interviewing experts was deemed as acceptable approximation and valuable supplementation to information from the literature, because experts revealed relevant and unpublished information. The expert elicitation was particularly valuable regarding the EQ-5D + C because the quality of life of patients with mercury intoxication had not been assessed previously. Open discussions of the HRQoL were preferred to individual expert assessments on order to base the final decision on the joint knowledge of the group. Applying a standardized instrument like the Delphi method was rejected due to the limited timeframe. However, the open discussion could have promoted consensuses and could have prevented the expression of ideas or possible disagreements which probably would have occurred, if the opinions were written and anonymous. The experts pointed out ten symptoms not mentioned in the reviews (Fig. 3). Particularly interesting is the information from the interview, that salivation might be substituted by a contrary symptom (dry mouth) in very severe cases. Due to the predefined inclusion criteria those symptoms were not included in the disease profile, but indicated the need for further research to study the association between CMMVI and the symptoms identified by the experts. Furthermore, in some cases the experts consulted yielded information on the probability of symptom occurrence or severity. Thus, *social avoidance* was included in the profiles as a symptom of severe cases (Table 1).

Using proxies to determine the HRQoL has been done before, e.g., relatives, friends, or professional caregivers were questioned if patients themselves were unable to answer the questionnaire due to illness or insufficient cognitive abilities (e.g., critically ill patients [55], patients with dementia [56], or young children [57, 58]). While the agreement between patients and proxies was relatively good in some studies (e.g., [55]), there is evidence that proxies tend to report a lower quality of life than the patients themselves [59, 60]. In future studies, the project results should be confirmed by involving a sufficient number of affected people to determine the patients’ reported outcome (PRO) [61].

Having the HRQoL determined by relatively few experts is a limitation of the project, however, it is a common approach. A comprehensive review including 154 HRQoL studies identified that in more than the half of the studies the assessment was done by experts or even by the authors themselves. The sample size ranged from one to 2579 respondents, while the maximum panel size of experts was 140 [62]. HRQoL assessment by comparatively few experts was also observed in a specific review of liver diseases involving 30 studies. With one exception (n < 100), the expert panels consisted of four to seven individuals [63]. The small panels may be a result of the limited number of experts on specific topics. An absolute guideline about the appropriate number of experts is not available, while a symposium on expert judgment policy in risk and environmental studies recommended of six to 12 experts [31, 64]. In the current study, it was possible to include five experts, which is close to the recommendation.

The EQ-5D + C assessment confirmed the differentiation of CMMVI into two stages due to the varying HRQoL. The resulting EQ-5D + C codes, 121222 for moderate and 233333 for severe cases, are comparable to assessments of other diseases. In the Dutch Disability Weight study, 175 disease stages were evaluated using the EQ-5D + C protocol [24]. No disease stage of the Dutch study has the same EQ-5D + C code as a moderate CMMVI case. Nevertheless, the criteria *self-care, pain, anxiety*, and *cognition* of a moderate CMMVI case are evaluated similar to the intermediate stage of Parkinson’s disease in the Dutch study. Indeed, the other two criteria, *mobility* and *usual daily activities*, were considered more severe in Parkinson’s disease (code 223222) than in a moderate CMMVI case (121222). A severe CMMVI case is assessed using the same code as schizophrenia at the stage *several psychotic episodes, severe and increasing permanent impairments* in the Netherlands [24].

Interesting is a comparison with the EQ-5D + C evaluation of alcoholism. In a previous attempt to estimate the BoD due to chronic mercury intoxication, the missing DW was replaced by a provisional DW of a comparable disease. The DW for alcoholism was chosen because alcoholism, like chronic mercury intoxication, is triggered by a substance, it shows comparable health symptoms, and it is a chronic condition [9]. In the Dutch DW study, the EQ-5D + C code for manifest alcoholism was assessed with 113221 [24]. The criteria *mobility, pain/discomfort,* and *anxiety/depression* are given values equal to a moderate case of CMMVI. However, the criteria, *self-care* and *cognition*, seemed to be greater problems in CMMVI while *usual activities* are highly restricted in patients with alcoholism and not in patients with moderate CMMVI.

The EQ-5D is brief and simple. The questionnaire is among the leading generic measures of HRQoL [65]. The sixth dimension, *cognition*, was added to the initial five dimensions of the EuroQol questionnaire – as was done in other studies (e.g. [24]) – to describe the components of CMMVI affecting cognitive aspects. Both moderate and severe cases show cognitive problems, confirming the choice of including the additional dimension.

As a result of the exclusion criteria defined, a small number of selected review articles were included, which might limit the project results. Even though, a full review of original articles might yield an even broader view on the symptoms of CMMVI, this would go far beyond the scope of the current assessments and would not be helpful to condense the available evidence. The profiles include only common symptoms and no case exceptions.

## Conclusions

Chronic exposure to metallic mercury vapor causes a range of symptoms. Based on knowledge obtained from experts and selected literature, disease profiles were set up for moderate and severe cases of CMMVI, including common symptoms and assessments of the HRQoL. Further steps, such as deriving DWs and conducting epidemiological data surveys, are needed to improve ascertainment and reporting of CMMVI, which is a requirement for developing and introducing prevention strategies to protect the workers’ health.

## Additional file

Additional file 1: 
**File S1.** Agenda of the expert meeting on December 14, 2012, in Ljubljana, Slovenia. ** File S2.** List of references used in the presentation *Chronic Hg intoxication in Idrija Mercury Mine.* **File S3.** Fields of research of the meeting participants. **File S4.** Interview questions. **File S5.** Complete list of body systems affected, symptoms, synonymous terms, and the decision whether to include or exclude in the disease profiles. (PDF 192 kb)

## Abbreviations

ASGM: artisanal small-scale gold mining
BoD: burden of disease
CMMVI: chronic metallic mercury vapor intoxication
DALY: disability-adjusted life years
DW: disability weight
DiWIntox-1: first part of the project *disability weights for chronic mercury intoxication*
EQ-5D + C: five EuroQol dimensions accompanied by the cognition add-on questionnaire
HRQoL: health-related quality of life
PRO: patients reported outcome

## References

[1] UNEP (2013). Minamata Convention on Mercury.

[2] Larson HJ (2014). The Minamata Convention on Mercury: risk in perspective. Lancet.

[3] UNEP (2013). Mercury: Time to act.

[4] Seccatore J, Veiga M, Origliasso C, Marin T, De Tomi G (2014). An estimation of the artisanal small-scale production of gold in the world. Sci Total Environ.

[5] WHO, UNEP (2008). Guidance for identifying populations at risk from mercury exposure.

[6] WHO (2013). Mercury Exposure and Health Impacts among Individuals in the Artisanal and Small-Scale Gold Mining (ASGM) Community. Preventing Disease through Healthy Environments.

[7] ILO (1999). Social and labour issues in small-scale mines. Report for discussion at the Tripartite Meeting on Social and Labour Issues in Small-scale Mines.

[8] ILO (2013). The prevention of occupational diseases: 2 million workers every killed year.

[9] Steckling N, Bose-O’Reilly S, Pinheiro P, Plass D, Shoko D, Drasch G (2014). The burden of chronic mercury intoxication in artisanal small-scale gold mining in Zimbabwe: data availability and preliminary estimates. Environ Health.

[10] Harris J, McCartor A (2011). Blacksmith Institute’s The World’s Worst Toxic Pollution Problems. Report 2011.

[11] Poulin J, Gibb H. Mercury: Assessing the environmental burden of disease at national and local levels In WHO Environmental Burden of Disease Series No 16 (Prüss-Üstün A ed.; 2008.

[12] Chatham-Stephens K, Caravanos J, Ericson B, Sunga-Amparo J, Susilorini B, Sharma P (2013). Burden of disease from toxic waste sites in India, indonesia, and the Philippines in 2010. Environ Health Perspect.

[13] Pruss-Ustun A, Vickers C, Haefliger P, Bertollini R (2011). Knowns and unknowns on burden of disease due to chemicals: a systematic review. Environ Health.

[14] Prüss-Üstün A, Mathers C, Corvalán C, Woodward A (2003). Introduction and methods: Assessing the environmental burden of disease at national and local levels. WHO Environmental Burden of Disease Series, No. 1.

[15] Murray CJL, Lopez AD (1996). Global Burden of Disease: A Comprehensive Assessment of Mortality and Disability from Diseases, Injuries, and Risk Factors in 1990 and Projected (Global Burden of Disease and Injury Series).

[16] Das P, Samarasekera U (2012). The story of GBD 2010: a “super-human” effort. Lancet.

[17] Banham D, Hawthorne G, Goldney R, Ratcliffe J (2014). Health-related quality of life (HRQoL) changes in South Australia: comparison of burden of disease morbidity and survey-based health utility estimates. Health Qual Life Outcomes.

[18] WHO. The Global Burden of Disease: 2004 update. Geneva: World Health Organization; 2008.

[19] Vos T, Flaxman AD, Naghavi M, Lozano R, Michaud C, Ezzati M (2012). Years lived with disability (YLDs) for 1160 sequelae of 289 diseases and injuries 1990–2010: a systematic analysis for the Global Burden of Disease Study 2010. Lancet.

[20] Schwarzinger M, Stouthard ME, Burstrom K, Nord E (2003). Cross-national agreement on disability weights: the European Disability Weights Project. Popul Health Metr.

[21] Stouthard MEA, Essink-Bot M-L, Bonsel GJ: Disability weights for diseases. Modified protocol and results for a Western European region. Eur J Public Health. 2000;10:24-30.

[22] Salomon JA, Vos T, Hogan DR, Gagnon M, Naghavi M, Mokdad A (2012). Common values in assessing health outcomes from disease and injury: disability weights measurement study for the Global Burden of Disease Study 2010. Lancet.

[23] Mathers C, Heggenhougen K (2008). Global Burden of Disease. International encyclopedia of public health.

[24] Stouthard MEA, Essink-Bot M-L, Bonsel GJ, Barendregt JJ, Kramers PGN, Water HP (1997). Disability Weights for Diseases in the Netherlands.

[25] Kruijshaar ME, Hoeymans N, Spijker J, Stouthard ME, Essink-Bot ML (2005). Has the burden of depression been overestimated?. Bull World Health Organ.

[26] Haagsma JA, van Beeck EF, Polinder S, Hoeymans N, Mulder S, Bonsel GJ (2008). Novel empirical disability weights to assess the burden of non-fatal injury. Inj Prev.

[27] Haagsma JA, Havelaar AH, Janssen BM, Bonsel GJ (2008). Disability adjusted life years and minimal disease: application of a preference-based relevance criterion to rank enteric pathogens. Popul Health Metr.

[28] van Spijker BA, van Straten A, Kerkhof AJ, Hoeymans N, Smit F (2011). Disability weights for suicidal thoughts and non-fatal suicide attempts. J Affect Disord.

[29] Haagsma JA, Polinder S, Cassini A, Colzani E, Havelaar AH (2014). Review of disability weight studies: comparison of methodological choices and values. Popul Health Metr.

[30] Essink-Bot ML, Pereira J, Packer C, Schwarzinger M, Burstrom K (2002). Cross-national comparability of burden of disease estimates: the European Disability Weights Project. Bull World Health Organ.

[31] Knol AB, Slottje P, van der Sluijs JP, Lebret E (2010). The use of expert elicitation in environmental health impact assessment: a seven step procedure. Environ Health.

[32] Slottje P, van der Sluijs JJ, Knol AB (2008). Expert Elicitation. Methodological suggestions for its use in environmental health impact assessments. Letter Report.

[33] Group EQ (1990). EuroQol--a new facility for the measurement of health-related quality of life. Health Policy.

[34] Oemar M, Oppe M (2013). EQ-5D-3 L User Guide. Basic Information on how to use the EQ-5D-3 L instrument.

[35] Krabbe PF, Stouthard ME, Essink-Bot ML, Bonsel GJ (1999). The effect of adding a cognitive dimension to the EuroQol multiattribute health-status classification system. J Clin Epidemiol.

[36] WHO (1976). Mercury. Environmental Health Criteria 1.

[37] WHO (1991). Inorganic Mercury. Environmental Health Criteria 118.

[38] WHO (2003). Elemental Mercury and Inorganic Mercury Compounds: Human Health Aspects. Concise International Chemical Assessment Document 50.

[39] ATSDR (1999). Toxicological Profile for Mercury.

[40] Nordberg G. 63. Metals: Chemical Properties and Toxicity - Mercury. In ILO Encyclopaedia of Occupational Health & Safety. Volume Fourth Edition. Edited by Nordberg G. Geneva: International Labour Office; 1998.

[41] Guzzi G, La Porta CA (2008). Molecular mechanisms triggered by mercury. Toxicology.

[42] Park JD, Zheng W (2012). Human exposure and health effects of inorganic and elemental mercury. J Prev Med Public Health.

[43] Clarkson TW, Magos L (2006). The toxicology of mercury and its chemical compounds. Crit Rev Toxicol.

[44] WHO. International Statistical Classification of Diseases and Related Health Problems 10th Revision (ICD-10 Version: 2010). Geneva: World Health Organization (WHO); 2010.

[45] Otto M, Ahlemeyer C, Tasche H, von Muhlendahl KE (1994). Mercury exposure. Nature.

[46] Wild CP (2005). Complementing the genome with an “exposome”: the outstanding challenge of environmental exposure measurement in molecular epidemiology. Cancer Epidemiol Biomarkers Prev.

[47] WHO. Mercury and health. Fact sheet N°361. World Health Organization; 2013.

[48] Eisler R (2003). Health risks of gold miners: a synoptic review. Environ Geochem Health.

[49] Boffetta P, Garcia-Gomez M, Pompe-Kirn V, Zaridze D, Bellander T, Bulbulyan M (1998). Cancer occurrence among European mercury miners. Cancer Causes Control.

[50] Boffetta P, Sallsten G, Garcia-Gomez M, Pompe-Kirn V, Zaridze D, Bulbulyan M (2001). Mortality from cardiovascular diseases and exposure to inorganic mercury. Occup Environ Med.

[51] Drasch G, Bose-O’Reilly S, Beinhoff C, Roider G, Maydl S (2001). The Mt. Diwata study on the Philippines 1999--assessing mercury intoxication of the population by small scale gold mining. Sci Total Environ.

[52] Rodrigues Filho S, dos Santos RLC, Villas Bôas RC, Castilhos ZC, Yallouz AV, Peregovich B, et al. Environmental and Health Assessment in two Small-Scale Gold Mining Areas - Indonesia. Final Report. Sulawesi and Kalimantan. Technical Final Report to UNIDO. 2004.

[53] Bose-O’Reilly S, Drasch G, Beinhoff C, Tesha A, Drasch K, Roider G (2010). Health assessment of artisanal gold miners in Tanzania. Sci Total Environ.

[54] Bose-O’Reilly S, Drasch G, Beinhoff C, Rodrigues-Filho S, Roider G, Lettmeier B (2010). Health assessment of artisanal gold miners in Indonesia. Sci Total Environ.

[55] Badia X, Diaz-Prieto A, Rue M, Patrick DL (1996). Measuring health and health state preferences among critically ill patients. Intensive Care Med.

[56] Gonzalez-Velez AE, Forjaz MJ, Giraldez-Garcia C, Martin-Garcia S, Martinez-Martin P (2015). Quality of life by proxy and mortality in institutionalized older adults with dementia. Geriatr Gerontol Int.

[57] Tarride JE, Burke N, Bischof M, Hopkins RB, Goeree L, Campbell K (2010). A review of health utilities across conditions common in paediatric and adult populations. Health Qual Life Outcomes.

[58] Kulpeng W, Sornsrivichai V, Chongsuvivatwong V, Rattanavipapong W, Leelahavarong P, Cairns J (2013). Variation of health-related quality of life assessed by caregivers and patients affected by severe childhood infections. BMC Pediatr.

[59] Crocker TF, Smith JK, Skevington SM (2015). Family and professionals underestimate quality of life across diverse cultures and health conditions: systematic review. J Clin Epidemiol.

[60] Nord E (1992). Methods for quality adjustment of life years. Soc Sci Med.

[61] Valderas JM, Ferrer M, Mendivil J, Garin O, Rajmil L, Herdman M (2008). Development of EMPRO: a tool for the standardized assessment of patient-reported outcome measures. Value Health.

[62] Tengs TO, Wallace A (2000). One thousand health-related quality-of-life estimates. Med Care.

[63] McLernon DJ, Dillon J, Donnan PT (2008). Health-state utilities in liver disease: a systematic review. Med Decis Making.

[64] Cooke R, Probst K. Highlights of the Expert Judgment Policy Symposium and Technical Workshop. Conference Summary. 2006.

[65] McDowell I. Measuring Health: A Guide to Rating Scales and Questionnaires, Third Edition. Oxford University Press; 2006.

