# Supplementary material for: Disease profile and health-related quality of life (HRQoL) using the EuroQol (EQ-5D + C) questionnaire for chronic metallic mercury vapor intoxication
Source: Health Qual Life Outcomes. 2015 Dec 9;13:196. doi: 10.1186/s12955-015-0388-0 (PMC4675011; doi:10.1186/s12955-015-0388-0)
Supplement: Additional file 1: — File S1. Agenda of the expert meeting on December 14, 2012, in Ljubljana, Slovenia. File S2. List of references used in the presentation Chronic Hg intoxication in Idrija Mercury Mine. File S3. Fields of research of the meeting participants. File S4. Interview questions. File S5. Complete list of body systems affected, symptoms, synonymous terms, and the decision whether to include or exclude in the disease profiles. (PDF 192 kb) [file 12955_2015_388_MOESM1_ESM.pdf]

Additional File 1

File S1: Agenda of the expert meeting on December 14, 2012, in Ljubljana, Slovenia

Topic	Presenter
1 Welcome and introduction of participants	All
2 Introduction of Jožef Stefan Institute	Milena Horvat
3 Hg exposure in Slovenia: recent studies at JSI	Ana Miklavcic
4 Chronic Hg intoxication in Idrija Mercury Mine	Alfred Kobal
5 Introduction of Bielefeld University	Nadine Steckling, Sonja Ramlow
6 Briefing for the interview	
7 Expert group interview	
8 The DiWIntox-project: Next steps	
9 Others	All

File S2: List of references used in the presentation *Chronic Hg intoxication in Idrija Mercury Mine*

ACGIH (2003), Albers et al. (1993), Andersen et al. (1993), Aschner (1997), Aschner (2000, 2007), ATSDR (1999), Barregård et al.(1994), Biernat et al. (1999), Burbur et al. (2006), Castoldi et al. (2001), Chapman et al. (1990), Clarkson and Magos (2006), DFG (2000), Ellingsen et al. 1993a,b, 2000, Erfurth et al. (1990), Falnoga 1995, Falnoga et al., 2000, 2002, Fawer et al. (1983), Gabrovec-Nahlk et al. (1977), Goldwater (1964), Hribernik (1950, 1955), Jonson and Montgomery (1997), Kobal (1965, 1975, 1991, 1994), Kobal et al. (1980, 1982, 1999, 2000, 2004, 2008), Kobal-Grum et al. (2006, 2010, 2012), Kosta et al. (1972, 1975), Kussmaul and Adolf (1861), Langolf et al. (1978), Lesky (1956), Letz et al. (2000), Longworth et al. (1995), Lucchini et al. (2003), Lund et al. (1993), MAC Committee (1969), Magos et al. (1978), Marsden (1978), Mathiesen et al. (1999), Meh and Kobal (2004), Netterstrom et al. (1996), OSHA (2007), Pfeifer (1989), Roels et al. (1985), Roels et al. (1989), Schaller et al. (1983), Scopoli (1754, 1761, 1771), Smith et al. (1983), Teleky (1912), Trahtenberg (1969), Tušek-Žnidarič et al. (2007), WHO (1976, 1980, 1991, 2003), Wood et al. (1973)

File S3: Fields of research of the meeting participants

Item	Interviewee 1	Interviewee 2	Interviewee 3	Interviewee 4	Interviewee 5
**University degree**	PhD Dr. med.	PhD Dr. med.	University PhD	PhD	PhD student
**Main research activity**	Occupational Hg exposure and health effects	Mercury, child, environment	Environmental science	Toxicology, Oncology	Food, science and technology; Hg exposure assessment
**Profession regarding mercury**	Professional (Medical view)	Professional (Medical and Public Health view)	Professional (Toxicological view)	Professional (Toxicological view)	Professional (Toxicological view)
**Type of profession***	Subject-matter expert	Subject-matter expert	Generalist	Subject-matter expert	Generalist
* The types of professions were predefined as follows: **Non-professional:** No professional knowledge; **Professional**: professional knowledge either as generalist, subject-matter expert, or normative expert. **Generalists:** "substantial knowledge" in the "discipline […] solid understanding of the context of the problem […] multidisciplinary"; **Subject-matter expert:** expert in the field, "essential for estimating subject-specific information"; **Normative experts**: "have knowledge, practical experience or skills that can support the elicitation process" [with reference to 1:7]					

File S4: Interview questions

List of guiding questions for the DiWIntox interview
(not all topics discussed are content of the current paper)

1. Please name synonyms for chronic mercury intoxication

2. Which terms of other mercury-induced diseases do not describe chronic mercury intoxication?

3. Which forms of mercury cause chronic mercury intoxication?

4. Which people are at risk of chronic mercury intoxication and which form of mercury are they exposed to?

Determination of the outcome: *In the following, the focus is on people suffering from chronic mercury intoxication who were chronically exposed to mainly elemental (metallic) mercury vapor.*

5. Which organs are affected by the determined outcome?

6. Please describe the symptoms of the determined outcome.

7. How long is the period between initial exposure to mercury and the first symptoms of the determined outcome?

8. Are there treatment options for the determined outcome?

9. Are there opportunities for mitigation, remission and/or complete clearance of the determined outcome?

10. Which conditions are necessary for mitigation of the determined outcome?

11. Are there opportunities for mitigation of the determined outcome in the following scenarios?

Scenario	Exposure	Treatment
1	constant	none
2	constant	treatment
3	minimzed	none
4	minimized	treatment
5	stopped	none
6	stopped	treatment

12. Is it possible to determine the period between symptoms onset and mitigation of the determined outcome, and if so, how long is it?

13. Which sequelae of the determined outcome are possible?

14. Is death a possible consequence of the determined outcome?

15. Is it possible – indeed, necessary – to distinguish the determined outcome in mild, moderate and severe?

16. Is the determined outcome different for men and women?

17. Is the determined outcome different for adults and children?

18. Please give a general description of the determined outcome.

Please indicate which statement best describes the health state of a person with the determined outcome.

19. Mobility
The person has no problems in walking about.
The person has some problems in walking about.
The person is confined to bed.

20. Taking care of oneself
Has no problems with self-care.
Has some problems washing or dressing themselves.
Is unable to wash or dress themselves.

21. Usual activities (e.g. work, study, housework, family, leisure activities)
Has no problems with performing their usual activities.
Has some problems with performing their usual activities.
Is unable to perform their usual activities.

22. Pain/Discomfort
Has no pain or discomfort.

	Has some pain or discomfort.<br>Has extreme pain or discomfort.
**23.**	Anxiety/Depression<br>Is no anxious or depressed.<br>Is moderately anxious or depressed.<br>Is extremely anxious or depressed.
**24.**	Cognition (e.g. memory, learning ability, concentration, comprehension)<br>Has no problems in cognitive functioning.<br>Has some problems in cognitive functioning.<br>Has extreme problems in cognitive functioning.
**25.**	Was the expert group interview suitable to develop an adequate and concise definition of the determined outcome in order to derive a disability weight for this condition?
**26.**	Were any gaps in data or knowledge found?
**27.**	Which gaps were found?

Supplementary File S**5:** Complete list of affected body systems, symptoms, synonym terms, and the decision for inclusion in the disease profiles

Row	Column A	Column B	Column C	Column D	Column E	Column F
				**Probability of occurrence (common or not common) and severity**		**Decision for disease profiles**
	**Affected body system / part** (alphabetically)	**Health symptom or sign and synonym terms** (alphabetically)	**Term used in DiWIntox** (if several synonyms do exist)	Text passages and corresponding references ["/" means: no text passages found]	Interpretation of the information from the text passages for DiWIntox	Exclusion (Excl.) or inclusion in moderate (Mod.) or severe case (Sev.)
**1**	**Dermal effects**	• dermatography [2]<br>• dermographism [P]<br>• marked dermographism [P]<br>• vasomotor disturbances such as uncontrolled blushing [P]	• dermographism	• "If excessive exposure is not corrected […] dermatography […] become more pronounced" [2].<br>• "increased Hg° absorption was frequently associated with […] marked dermographism" [P].	Rising severity as result of high ongoing exposure. Very few sources.	Excl.
**2**	**Endocrine Effects**	Signs<br>• decreased serum serotonin [P]<br>• higher level of melatonin [I]<br>• enhanced glutaminergic activity [P]<br>• enhanced dopaminergic activity [P]<br>• increased blood melatonin [P]<br>• lower level of serotonin [I]	• changing hormone level	/	Very few sources.	Excl. *[just mentioned in the DIWIntox meeting; not in the literature]*
**3**	**Effects of the digestive system**	Synonyms / summarizing symptom categories<br><br>• digestive disorders [2]<br>• digestive symptoms [2]	• digestive disorders	• "[…] digestive and nervous symptoms predominate and, although the former are of earlier onset, the latter are more obvious" [2].	Common; early sign; earlier than nervous symptoms.	
				• "If excessive exposure is not corrected […] perhaps, digestive disorders (stomatitis, diarrhoea) […]" [2] may occur.	Result of high ongoing exposure.	
				• „The main early signs include slight digestive disorders, in particular, loss of appetite […] " [2].	Early sign.	
**4**		• bluish (dis)coloration [I]<br>• coloration of the gingiva [I]<br>• coloration of the oral cavity [I]<br>• copperish colorated palatum [P]	• coloration of the oral cavity	• "[…] exposed to very high mercury concentration they can get […] coloration of the gingiva […] bluish coloration […]" [I]<br>• "[…] higher mercury concentrations or many time to high mercury concentration" "the oral cavity" is affected "[…] coloration of the […] gingiva […] bluish coloration" [I].	Result of high exposures. Very few sources.	Excl. *[just mentioned in the DIWIntox meeting; not in the literature]*
**5**		• changing taste [I]<br>• metallic taste [I]<br>• sweet metallic taste [P]	• changing taste	• Changing taste: main symptom [I].<br>• "increased Hg° absorption was frequently associated with […] sweet metallic taste" [P].	Common. Very few sources.	Excl. *[just mentioned in the DIWIntox meeting; not in*

				• "[…] the main symptoms are tremor, erethism and gingivitis […] coordination problems, then you have the […] metallic taste" [I].	Later than main symptoms.	*the literature]*
**6**		• damage to the lining of the mouth [3]<br>• disease affecting the oral cavity [I]<br>• inflammation in the mouth [I]<br>• ulcerations of the oral mucosa [3] [4]<br>• stomatitis [2] [3] [4] [5] [6]<br>• trouble with oral cavity [I]	• stomatitis	• "If excessive exposure is not corrected […] perhaps, […] stomatitis […]" [2] may occur.	Result of high ongoing exposure.	**Sev.**
				• "[…] stomatitis […] are also associated with high occupational exposure" [5], p. 348, [6], p. 619.<br>• "[…] exposed to very high mercury concentration they can get […] disease affecting the oral cavity" [I].<br>• "[…] higher mercury concentrations or many time to high mercury concentration" "the oral cavity" is affected [I].	Result of high exposure.	
				• "Damage to the lining of the mouth […] can also occur from exposure to lower levels of mercury vapor over longer periods […]." [3], p. 13.	Result of long and low exposure.	
**7**		• diarrhea [2] [3]		• "If excessive exposure is not corrected […] perhaps, […] diarrhea […]" [2] may occur.	Result of high ongoing exposure. Very few sources.	Excl.
**8**		• drooling [3] [4]<br>• salivation [I] [5] [6]	• salivation	• "[…] the main symptoms are tremor, erethism and gingivitis […] coordination problems, then you have the salivation […]" [I].	Later than the main symptoms	**Mod. & Sev.** (salivation / excessive salivation)
				• excessive salivation […] "associated with high occupational exposures" [5], p. 348, [6], p. 619.	Rising severity	
**9**		• dry mouth [I]		• "at initially and moderate is the salivation more effective […]" in severe cases "[…] it is not the salivation but […] dry mouth" [I].	Severe case. Very few sources.	Excl. *[just mentioned in the DIWIntox meeting; not in the literature]*
**10**		• gingivitis [I] [5] [6] [7] [8] [9]<br>• sore gums [3] [4]	• gingivitis	• „The major clinical features […] gingivitis" [5], p. 347<br>• "The classic symptoms […] gingivitis" [8].	Common	**Sev.**
				• "Gingivitis […] are also associated with high occupational exposure" [5], p. 348, [6], p. 619.	Result of high exposure	
				• "no evidence of […] gingivitis below a time-weighted occupational exposure to mercury in air of 100 µg/m^3" [9].<br>• "[…] exposed to very high mercury concentration they can get […] gingivitis" [I].<br>• "[…] higher mercury concentrations or many time to high mercury concentration" "the oral cavity" is affected "[…] gingivitis" [I].	Level of observation. No early sign.	

**11**		• anorexia [2]<br>• appetite loss [8]<br>• loss of appetite [P] [8] [9]	• loss of appetite	• „The main early signs include […] loss of appetite […] " [2].<br>• „Symptoms such as loss of appetite […] have also been found to occur at mercury levels below 100 µg/m^3" [9].<br>• „[…] loss of appetite […] have been reported to occur at mercury levels below 0.1 mg/m^3", before the classical symptoms erethism, tremor, gingivitis [8].<br>• "[…] a significant increase was observed at mercury concentrations in air of 0.06-0.1 mg/m^3 in […] loss of appetite […]" / "appetite-loss" [8].	Level of observation.<br>Early sign.	**Av. & Sev.** (appetite loss / anorexia)
				• "increased Hg° absorption was frequently associated with […] loss of appetite" [P].	Common	
				• „If excessive exposure is not corrected […] perhaps, […] deterioration in […] anorexia […]" [2] may occur.	Rising severity as result of high ongoing exposure	
**12**		• loss of teeth [I] [P]		/	Very few sources.	Excl. *[just mentioned in the DIWIntox meeting; not in the literature]*
**13**		• unspecified oropharyngeal symptoms [3]		/	Very few sources.	Excl.
**14**	**General effects**	<u>Synonyms / summarizing symptom categories</u><br>• deterioration in general status [2]		• „If excessive exposure is not corrected […] perhaps, […] deterioration in general status (anorexia, weight loss)" [2] may occur.	Rising severity as result of high ongoing exposure	
**15**		• fatigue [I] [P] [3] [4] [6] [9]<br>• lassitude/o [I] [P]	• fatigue	• "increased Hg° absorption was frequently associated with […] fatigue" [P].	Common	**Mod. & Sev.**
				• Fatigue: "negative findings at low exposure levels (0.025–0.076 mg/m^3)" [3].<br>• "Long-term, low-level exposure has been found to be associated with less pronounced symptoms of erethism, characterized by fatigue" [9].	Level of observation.<br>Result of low ongoing exposure.<br>No distinction in moderate and severe cases possible.	
**16**		• fever [7]		/	Very few sources.	Excl.
**17**		• headache [I] [4]		• "Prominent symptoms include […] headaches […]" [4], p. 22, (29).	Common.<br>Very few sources.	Excl.
**18**		• malaise [7]		/	Very few sources.	Excl.
**19**		• sweating [2]		• "If excessive exposure is not corrected, […] sweating […] become more pronounced" [2].	Rising severity as result of high ongoing exposure.<br>Very few sources.	Excl.

**20**		• weight loss [2] [8]		• "[…] a significant increase was observed at mercury concentrations in air of 0.06-0.1 mg/m^3 in […] weight loss […]" [8].	Level of observation. Early sign. Very few sources.	Excl.
				• „If excessive exposure is not corrected […] perhaps, […] deterioration in […] weight loss […]" [2] may occur.	Rising severity as result of high ongoing exposure. Very few sources.	
**21**	**Hematological effects**	• anaemia [2]		• "Chronic poisoning is accompanied by mild anaemia sometimes preceded by polycythaemia resulting from bone marrow irritation" [2].	Common; mild severity. Very few sources.	Excl.
**22**		• bone marrow irritation [2]		• "Chronic poisoning is accompanied by mild anaemia sometimes preceded by polycythaemia resulting from bone marrow irritation" [2].	Not common. Very few sources.	Excl.
**23**		• eosinophilia [2]		• "[…] eosinophilia have also been observed" [2].	Very few sources.	Excl.
**24**		<u>Signs</u> • higher level of glutathione [I]		/	Very few sources.	Excl. *[just mentioned in the DIWIntox meeting; not in the literature]*
**25**		<u>Signs</u> • changing catalase [I]		/	Very few sources.	Excl. *[just mentioned in the DIWIntox meeting; not in the literature]*
**26**		• lymphocytosis [2]		• "Lymphocytosis […] have also been observed" [2].	Not common. Very few sources.	Excl.
**27**		• polycythaemia [2]		• "Chronic poisoning is accompanied by mild anaemia sometimes preceded by polycythaemia resulting from bone marrow irritation" [2].	Not common. Very few sources.	Excl.
**28**	**Immunological and lymphoreticular effects**	• immune response [9] • affected immune system resulting in a decreased resistance to infection or cancers [5] • immune dysregulation that can induce the development of allergy or autoimmunity [5] • immunological changes [I]	• immunological changes	/	No distinction in moderate and severe cases possible.	**Mod. & Sev.**
**29**		• Increase in total IgE in serum [4]	• changes of immune parameters	/	Very few sources.	Excl.
**30**		• increase in anti-DNA antibodies [4]		/	Very few sources.	Excl.

**31**	**Metabolic effects**	Signs<br>• oxidative stress [I]	/	Very few sources.	Excl.<br>*[just mentioned in the DIWIntox meeting; not in the literature]*
**32**	**Neurological effects**	Synonyms / summarizing symptom categories<br>• effects on nervous system [6]<br>• frank neurotoxicity [3]<br>• impairment of the central nervous system [I]<br>• neurological changes [I] [P]<br>• neurological effects [3] [9]<br>• neurological manifestations [2]<br>• nervous symptoms [2]<br>• nervous system damage [6]<br>• nervous system disorders [3]<br>• other signs of neurotoxicity [3] [4]<br>• symptoms of the central nervous system [8]<br>• subclinical, peripheral neuropathy [P]	• Central nervous system: "[…] major target organ […]" [3], p. 33, [2]; "[…] notable target organ […]", [5], p. 347; "[…] the most sensitive target" [4], p. 29; "[…] the critical organ […]" [I] [8] [9].<br>• "[…] nervous symptoms predominate" [2].	Common	
			• "Nervous system disorders […] consistent and pronounced" [3], p. 58.	Occurs in moderate and severe cases	
			• "[…] digestive and nervous symptoms […] although the former are of earlier onset, the latter are more obvious" [2].	Occurs later than digestive	
			• „Slight renal involvement […] may be detectable earlier than neurological involvement" [2].	Occurs later than renal involvement	
			• "[…] subtle effects on the central nervous system" [….]. Renal changes have been observed at somewhat higher exposure levels" [4], p. 30.<br>• "Effects on the kidney […] have been reported but only at doses higher than those associated with the onset of signs and symptoms from the central nervous system" [8].	Occurs in lower exposures than kidney/renal changes	
			• "Until recently […] effects […] on the kidney had been reported only at doses higher than those associated with the onset of signs and symptoms from the central nervous system. New studies have, however, reported kidney effects at lower exposure levels" / "[…] effects […] on the kidney had been reported only at doses higher than those associated with the onset of CNS signs and symptoms. Since then several new studies have been carried out, and kidney effects have been seen at lower exposure levels" [9].	Occurs in higher exposures than kidney effects	
			• "[…] adverse effects in other organs […] occur at exposure levels higher than those affecting the central nervous system […]" [4], p. 30.	Occurs in lower exposures than in other organs	
			• "If excessive exposure is not corrected, neurological […] manifestations (e.g., tremor, sweating, dermatography) become more pronounced" [2].	Rising severity as result of high ongoing exposure	
			• Central nervous system: "[…] severity [of effects] increases" / "[…] the symptoms may intensify and/or become irreversible as exposure duration and/or concentration	Rising severity as result of ongoing and/or rising	

				increase" [4], p. 29f.<br>• Neurological effects: "Symptoms intensify and may become irreversible as exposure duration and/or concentration increases" [3], p. 58.	exposure		
**33**		• abnormal nerve conduction velocities [3] [4] [6]<br>• abnormalities in sensory and peripheral nerve conduction [5]<br>• adverse effects of peripheral nerve function [P] [3]<br>• decreased nerve conduction [3] [4]<br>• decreased nerve conduction velocity [9]<br>• lower sensory-motor conduction velocities of the ulnar median nerve [P]<br>• effects on peripheral (neuro)system [I]<br>• peripheral nerve involvement [9]<br>• peripheral nerve abnormalities [3] [5]<br>• slowed sensory and motor nerve conduction velocities [3] [4]	• peripheral nerve abnormalities	• "Prominent symptoms include […] polyneuropathy ([…] slowed sensory and motor nerve conduction velocities) […]" [4], p. 22.	Common	*Contradiction*	**Mod.**
				• "[…] peripheral nerve abnormality can present but is not common" [5], p. 348.	Not common		
				• "[…] peripheral nerve function […] may be associated with very low exposures" [3], p. 61.<br>• Peripheral nerve function: […] adverse effects may be associated with very low exposures" [4], p. 22f.	Result of low exposure		
				• motor and sensory nerve conduction velocities: „correlations between exposure level or duration and effects" [2].	Rising severity		
				• "[…] dose-response relationship between urine mercury concentrations above 50 µg/litre and nerve conduction tests" [9].	Level of observation.		
**34**		• axonal sensor motor polyneuropathy [7]<br>• Neurological neuropathy [I]<br>• peripheral neuropathy [9]<br>• polyneuropathy [3] [4] [6]	• polyneuropathy	• "Prominent symptoms include […] polyneuropathy (paraesthesia, stocking-glove sensory loss, hyperactive tendon reflexes, slowed sensory and motor nerve conduction velocities) […]" [4], p. 22, (29).	Common.		**Sev.**
				• "[…] subjects with reported clinical polyneuropathy had significantly higher peak levels of mercury in urine than the subjects without those signs" [9].	Result of high exposure.		
				• Polyneuropathy: "correlations between exposure level or duration and effects" [3].	Rising severity.		
				• "peripheral neuropathy at urinary levels of 50-100 µg/litre" [9].	Level of observation.		
**35**		• brisk reflexes [I] [P]<br>• hyperactive tendon reflexes [3] [4]<br>• reflex abnormalities [3]	• reflexes abnormalities	• "Prominent symptoms include […] polyneuropathy ([…] hyperactive tendon reflexes […]) […]" [4], p. 22.	Common.		**Mod.**
				• Reflex abnormality: "correlations between exposure level or duration and effects" [3].	Rising severity.		
**36**		• paraesthesia**,** paresthesia [3] [4]	• paresth	• "Prominent symptoms include […] polyneuropathy (paraesthesia […]) […]" [4], p. 22.	Common.<br>Very few sources.		Excl.

				esia			
**37**			• decreased sensation [6]<br>• hypersensitivity [9]<br>• reduced distal sensation [6]<br>• sensory disturbances [4]<br>• stocking-glove sensory loss [4]<br>• worse vibration sense [6]	• sensory disturbances	• "Prominent symptoms include […] polyneuropathy ([…] stocking-glove sensory loss […]) […]" [4], p. 22.	Common.	**Mod.**
**38**			• neurotic disorders [2]		• "The main early signs include […] neurotic disorders varying in intensity" [2].	Common; rising severity.<br>Very few sources.	Excl.
**39**			• prolongation of brainstem auditory-evoked potentials [3] [4]<br>• prolonged somatosensory-evoked potentials [3] [4]<br>Signs<br>• electroencephalographic changes [3]<br>• slower and more attenuated electroencephalograms (EEGs) [4]	• evoked potentials	• electroencephalographic changes: "correlations between exposure level or duration and effects" [3].	Rising severity.<br>Very few sources.	Excl.
**40**		**Neurological effects regarding the musculoskeletal system**	Synonyms / summarizing symptom categories<br>• neuro-muscular/ neuromuscular changes [2] [4]<br>• motor disturbance/s [I] [4]<br>• motor system disturbances [3]<br>• psychomotor changes [2]<br>• psychomotor dysfunction [9]<br>Signs<br>• performance deficits in tests of motor function [4]<br>• test for psychomotor skills [4]		• "Prominent symptoms include […] neuromuscular changes changes (weakness, muscle atrophy, muscle twitching, electromyographic abnormalities) […]" [4], p. 22, (29).<br>• motor disturbance/s: main symptom [I].	Common	
					• "Recent studies using sensitive tests for psychomotor skills […] suggest that adverse effects may be associated with very low exposures." [4], p. 22f.<br>• […] effects of "psychomotor skills […] may be associated with very low exposures" [3], p. 61.	Result of low exposure	
					• "[…] preclinical psychomotor dysfunction related to the central nervous system occurs when blood mercury levels rise to values between 10 and 20 µg/litre and when mercury in urine exceeds 50 µg/g creatinine" [9].	Level of observation.	
**41**			• acceleration tremor [6]<br>• intention(al) tremor [P] [5] [6] [8] [9]<br>• intermittent tremor [2]<br>• postural tremor [6]<br>• resting tremor [5]<br>• tremor(s) [I] [P] [2] [3] [4] [5] [6] [7]	• tremor	• Tremor: "one of the most characteristic features" [8]; "prominent symptom", [4], p. 22, (29); „the major clinical features […]" [5], p. 347; "the main feature" [6], p. 619.<br>• "Tremor is considered to be the early neurological sign […] which presents intentional tremor or resting tremor, or both." [5], p. 347.<br>• "objective tremor": "classical signs and symptom" [8].	Common	**Mod. & Sev.**

			[8] [9], affecting arm [I], entire body [P], eyelids [8], finger [I] [P] [8], feed [P], hands [P] [3] [9], head [P], lips [8], protruding tongue [8], whole body [I]<br><br>Signs<br>• alterations in the steadiness of the handwriting [8]<br>• characteristic appearance of handwriting (reflects tremor) [9]<br>• changing handwriting [P]		• "intentional tremor": "classic symptom" [8].<br>• "The main early signs include […] intermittent tremor, sometimes in specific muscle groups" [2].<br>• Tremor: main symptom; most important [I].<br>• "increased Hg° absorption was frequently associated with […] fine finger tremor" [P].			
					• "[…] increased prevalence of tremor was apparent in […] the groups with the shortest exposure duration (1-4 years)" [9].<br>• "[…] the most important […] the most pronounced [symptom] is slowly, slowly tremor" [I].<br>• "initial fine finger tremor" [P].	Early sign		
					• "Tremor […] follows the minor psychological disturbances […] insomnia, shyness, nervousness, and dizziness", [8].	Occurs later than neuropsychological disturbances		
					• "[…] tremor and erethism come together" [I].	Occurs together with erethism	*Contradiction*	
					• „Occupational exposure has resulted in erethism […]. With continuing exposure, a fine tremor develops […]" [9].	Occurs later than erethism		
					• "[…] increased prevalence of tremor was apparent in […] the groups with the lowest exposure (urine mercury level of 5-50 µg/g creatinine) […] [9].<br>• "[…] tremor […] has been observed at low urine concentrations (down to 25-35 µg/g creatinine)" [9].<br>• "[…] tremor […] may be associated with very low exposures" [3], p. 61, [4], p. 22f.	Occurs in low exposures		
					• "tremor develops gradually (i) initially in the form of fine finger trembling, (ii) then spread to the limbs showing higher aplitude which may be interrupted by coarse shaking movements, (iii) finally in heavy intoxications may spread to the other parts of the body" [P].<br>• "tremors (which may be mild or severe depending on the degree of exposure)" [3], p. 61.<br>• "increased tremor" [6].<br>• "pronounced tremor" [3].<br>• "With continuing exposure […] the tremor develops gradually in the form of fine trembling of the muscles interrupted by coarse shaking movements every few	Rising severity (with duration of exposure)		

					minutes […]" [8].<br>• „increase in the frequency of objective tremors" [8].<br>• "[…] significant increase in average tremor frequency with elevated urinary mercury level" [9].<br>• Tremor: "The highest peak frequency of the acceleration (i.e., the frequency corresponding to the highest acceleration) […] was significantly related to duration of exposure and age" [4], p. 23.<br>• "Mercury-induced tremor in milder cases is intentional, which occurs during guided movements (finger-to-nose test), but in more severe cases tremor becomes postural (tremor in the extended arm)" [6].<br>• "If excessive exposure is not corrected, […] tremor […] become more pronounced" [2].<br>• "Initially […] slight tremor" [2].<br>• "initially tremor is usually fine tremor" [I].<br>• "with high tremor this is not possible to eat, they have trouble […] to drink and they […] cannot sleep" [I].<br>• „correlations between exposure level or duration and effects" [3].<br>• "Dramatic alterations in the steadiness of the handwriting may be seen in persons suffering from mercurial tremor" [8].		
					• "A significant increase in the frequency of objective tremors was noted at mercury levels in air above 0.1 mg/m^3 in agreement with previous reports on occupational exposure" [8].<br>• „objective tremors […] expected to appear after chronic exposure of workers to air concentrations of mercury above 0.1 mg/m^3" [8].<br>• "significant increase in average tremor frequency […] was observed at urine concentrations above about 50 µg/litre" [9].<br>• "no evidence of […] intentional tremor […] below a time-weighted occupational exposure to mercury in air of 100 µg/m^3" [9].<br>• "At a urinary mercury excretion level of 100 µg per g creatinine, the probability of developing […] tremor […] is high" [9].<br>• "[…] frank neurotoxicity (pronounced tremors […]) was generally observed at >300 µg mercury in a 24-hour urine […] or at >0.1 mg/m^3 […]" [3], p. 61.	Level of observation.	

					• "high elevated tremor, with muscles in the face" [I].<br>• Tremor: "is more pronounced in intentional movements […] trouble eating and drinking" [I].	Rising severity includes other parts of the body	
					• "fine tremor", […] first "in the finger and then go to the arm and then the whole body, […] they make some grimace […] and […]is difficult to speak" [I].<br>• "high elevated tremor, with muscles in the face" [I].<br>• "fine tremor, initially involving the hands" [9].<br>• "intensive tremor of the hands, head, feed, and the entire body" [P].<br>• Tremor: "[…] initially affecting the hands and sometimes spreading to other parts of the body" [4], p. 22, (29).<br>• Tremor: "It may be seen in the fingers, but also on the closed eyelids, lips, and on the protruding tongue" [8].<br>• „[…] a fine tremor develops, initially involving the hands and later spreading to the eyelids, lips, and tongue, causing violent muscular spasms in the most severe cases" [9].<br>• "The main early signs include […] intermittent tremor, sometimes in specific muscle groups" [2].<br>• „[…] mainly limb tremor that can spread to the tongue and face muscles" [I].<br>• "Tremor is also seen on the closed eyelids, on the lips and on the protruding tongue" [P].	Occurs in different body parts	
**42**			• ataxia [I] [P]<br>• coordination problems [I]<br>• decreased coordination [6]<br>• discoordination [I]<br>• effects on psychomotor coordination [3]<br>• impaired coordination [6]<br>• impaired coordination ability [P]<br>• muscle incoordination [3]<br><u>Signs</u><br>• decrement of arm-hand steadiness [4]<br>• electromyographic abnormalities [3] [4]<br>• performance decrements in psychomotor skills (e.g. finger tapping, reduced hand-eye coordination) [3] [4]<br>• reduced hand-eye coordination [3]	• coordination problems	• ataxia: main symptom [I].<br>• "Prominent symptoms include […] electromyographic abnormalities […]" [4], p. 22.<br>• Coordination problems: main symptom [I].	Common.	**Mod. & Sev.** (coordination problems / severe coordination problems)
					• Psychomotor coordination: „correlations between exposure level or duration and effects" [3].<br>• electromyographic abnormalities: "correlations between exposure level or duration and effects" [3].	Rising severity with exposure level and duration.	

				[4]<br>• abnormal Romberg test [4]				
**43**				• changes in control of locomotor function [P]<br>• coarse shaking movements [8]<br>• dysfunction of movement control [P]<br>• periodic contractile movements of legs [P]<br>• unsteady walking [3] [4]<br>• worse motor speed [6]<br><u>Signs</u><br>• difficulty with heel-to toe-gait [3] [4]	• dysfunction of movement control	/	No distinction in moderate and severe cases possible	**Mod. & Sev.**
**44**				• dysdiadochokinesis [4]		/	Very few sources.	Excl.
**45**				• muscle atrophy [4]		• "Prominent symptoms include […] muscle atrophy […]" [4], p. 22.	Common.<br>Very few sources.	Excl.
**46**				• muscle cramps [3] [4]		/	Very few sources.	Excl.
**47**				• muscle fasciculations [3] [4]		/	Very few sources.	Excl.
**48**				• muscle pain [3] [4]		/	Very few sources.	Excl.
**49**				• muscular spasms [9]		• „Tremor […] causing violent muscular spasms in the most severe cases" [9].	Muscular spasms in the most severe cases of tremor.<br>Very few sources.	Excl.
**50**				• muscle twitching [4]<br>• myoclonus [3] [4]	• myoclonus	• "Prominent symptoms include […] muscle twitching […]" [4], p. 22.	Common.<br>Very few sources.	Excl.
**51**				• trembling [P]<br>• trembling of the muscles [8]	• trembling	• "the tremor develops gradually in the form of fine trembling of the muscles" [8].	Beginning of tremor.<br>Very few sources.	Excl.
						• "heavy trembling of the arms and whole body" in high exposure [P].	Severe case.	
**52**				• hyper impairment [I]<br>• impairment [I]<br>• weakness [3] [4]	• weakness	• "Prominent symptom" […] weakness […]" [4], p. 22.	Common.<br>No distinction in moderate and severe cases possible.	**Mod. & Sev.**
**53**		**Neurological effects**	**auricular syste**	• deafness [3]		/	Very few sources.	Excl.

		regardi ng other system s	m					
**54**			ocula r syste m	• blurred vision [3] [4]<br>• changes in vision (construction (or narrowing) of the visual field) [3]<br>• defect in visual evoked response [6]<br>• difficulty seeing [3]<br>• restriction of visual fields [3]	• difficulty seeing	• "[…] restriction of visual fields, difficulty seeing […] was generally observed at >300 µg mercury in a 24-hour urine […] or at >0.1 mg/m^3 […]." [3], p. 61.	Level of observation. Result of high exposures (in comparison to other levels mentioned).	**Sev.**
**55**			speec h	• tremulous speech [3] [4]		/	Very few sources.	Excl..
**56**		**Neurologica l effects in terms of behavioral, cognitive, emotional, mental effects**		Synonyms / summarizing symptom categories<br>• change of personality [I]<br>• changes in behavior [2]<br>• changes in control of behavior [P]<br>• changes in control of emotions [P]<br>• changing / changes in personality traits [I] [P]<br>• changing personality [I]<br>• cognitive disturbances [4]<br>• cognitive impairments [3]<br>• emotional changes [3]<br>• emotional lability [3] [4]<br>• mental disturbances [8]<br>• neurobehavioural impairment [9]<br>• personality changes [3]<br>• personality disorders [2]<br>• personality disturbances [4]<br>• psychiatric disturbances [8]<br>• psychological disturbances [6] [8] [9]<br>• psychological effects [8]<br>• psychological impairment [I]<br>• neurocognitive disorders [7]		• Psychological disturbances: "main feature" [6], p. 619; "major clinical feature" [5], p. 347.<br>• Emotional lability: "prominent symptom" [4], p. 22, (29).<br>• Changing personality: main symptom [I].	Common	
						• "If excessive exposure is not corrected, […] changes in behaviour and personality disorders" [2] occur.	No early sign	
						• "[…] minor psychiatric disturbances such as insomnia, shyness, nervousness, and dizziness in workers exposed to elemental mercury vapour concentrations of the order of 0.1 mg/m^3" [8].<br>• Psychological disturbance "[…] occur at mercury levels below 100 µg/m^3" [9]; "may be seen at air concentrations of mercury below 0.10 mg/m^3" [8].	Level of observation.	
						• Emotional changes: "correlations between exposure level or duration and effects" [3].	Rising severity	
**57**				• anger [3] [4]		/	Very few sources.	Excl.
**58**				• anxiety [6] [8]		• "the most commonly reported syndrome includes […] anxiety" [8].	Very few sources.	Excl.
**59**				• avoidance of public places and friends [P]<br>• contact with people is problematic [I]<br>• desire to remain unobserved an unobtrusive [6]<br>• introverted [P]	• social avoidance	• "the contact with the people is more problematic in heavy mercury intoxication" [I].	Severe case	**Sev.**

**60**			• bad (labile) temper [I] [P]<br>• depression [5] [8] [9]<br>• depressive [I] [P]<br>• depressive feelings [9]<br>• depressive mood [I] [P]<br>• manic-depressive psychoses [8]<br>• suicidal melancholia [8]	• depressive mood	• „[…] the most commonly reported syndrome includes […] depression" [8].	Common	**Mod. & Sev.** (depressive mood / depression)
					• "The heavy mercury intoxication, the depressive mood is more pronounced" [I].	Rising severity	
					• "In the most severe cases […] suicidal melancholia, or even manic-depressive psychoses […]" [8].	Severe case	
**61**			• confidence loss [3] [4]<br>• loss of self-confidence [6] [8]<br>• negative self-concept [P]	• loss of confidence	• "Prominent symptoms include […] confidence loss […]" [4], p. 22.<br>• "the most commonly reported syndrome includes […] loss of self-confidence" [8].	Common. No distinction in moderate and severe cases possible.	**Mod.**
**62**			• confusion [3] [4]		/	Very few sources.	Excl.
**63**			Signs<br>• decreases in performance on tests that measured intelligences (similarities test) [3]<br>• disturbances in tests on verbal intelligence [4]	• decreasing intelligence	• "Decreases in performance on tests that measured intelligence (a similarities test) […] were observed in chloralkali workers exposed for an average of 16.9 years to low levels of mercury […]" [3], p. 63.	Result of long and low exposure. Very few sources.	Excl.
**64**			• delirium [8]<br>• delusions [7]<br>• hallucinations [7] [8]	• delirium	• „In the most severe cases delirium with hallucinations, suicidal melancholia, or even manic-depressive psychoses have been described" [8].	Severe case. Very few sources.	Excl.
**65**			• difficulties with memory [3]<br>• impaired (short term) memory [P]<br>• loss of memory / memory loss [3] [4] [7] [8] [9]<br>• memory deficits [3]<br>• memory disturbances [3] [4] [6] [9]<br>• memory impairment [5]<br>• memory problems [I]<br>• short-term memory deficits [3] [9]<br>Signs<br>• decreases in performance on tests that measured memory (digit span and visual reproduction tests) [3]<br>• disturbances in tests on memory [4]	• memory impairments	• "Prominent symptoms include […] memory loss […]" [4], p. 22, (29).<br>• "The classic symptoms […] loss of memory[…]" [8].<br>• "the most commonly reported syndrome includes loss of memory" [8].	Common	**Mod. & Sev.** (memory impairments / memory loss)
					• "Decreases in performance on tests that measured […] memory (digit span and visual reproduction tests) were observed in chloralkali workers exposed for an average of 16.9 years to low levels of mercury […]" [3], p. 63.	Result of long and low exposure	
					• "effects on short-term memory […] The severity of the effects was found to be related to the intensity of mercury exposure" [9].	Severity rises with exposure	
					• "loss of memory", "memory loss" [3] [4] [7] [8] [9].<br>• "at heavy intoxication […] especially the short term memory" is affected [I].<br>• "impaired memory (short-term memory) at heavy intoxications" [P].<br>• Memory deficits: „correlations between exposure level or	Severe case (memory loss)	

					duration and effects" [3].		
**66**			• difficulty in concentration [5]<br>• poor concentration [3] [4]<br><u>Signs</u><br>• effects on cognitive skills [3] [4]<br>• performance deficits in tests of cognitive function [4]	• difficulty in concentration	• "Prominent symptoms include […] performance deficits in tests of cognitive function […]" [4], p. 22, (29).	Common. No distinction in moderate and severe cases possible.	**Mod. & Sev.**
**67**			• diffidence [5] [6]<br>• shyness [I] [P] [3] [4] [5] [6] [7] [8] [9]<br>• timidity [5] [6]<br>• timidness [P]	• shyness	• "Prominent symptoms include […] excessive shyness […]" [4], p. 22; "excessive shyness […] as the principal features" [9].	Common.	**Mod. & Sev.** (shyness / extreme shyness)
					• "Tremor […] follows […] shyness […]" [8].	Occurs earlier than tremor	
					• "increasing shyness" [5] [6].<br>• "excessive shyness" [3] [4] [9].<br>• "extreme shyness" [7].<br>• "excessive timidity" [5] [6].	Rising severity	
					• "minor psychiatric disturbances such […] shyness […] elemental mercury vapour concentrations of the order of 0.1 mg/m^3" [8].<br>• "[…] a significant increase was observed at mercury concentrations in air of 0.06-0.1 mg/m^3 in […] shyness" [8].	Level of observation.	
**68**			• dizziness [8]		• "Tremor […] follows […] dizziness" [8].	Occurs earlier than tremor.<br>Very few sources.	Excl.
					• dizziness occurs in workers "[…] exposed to elemental mercury vapour concentrations of the order of 0.1 mg/m^3" [8].	Level of observation.	
**69**			• drowsiness [8]<br>• Frequent awakenings [P]<br>• insomnia [I] [3] [4] [6] [7] [8] [9]<br>• somnolence [5]<br>• sleep disorder [I] [P] [3] [4]<br>• sleeping problems / problem with sleep [I] [P]<br>• bad dreams [P]<br>• vivid dreams [9]	• sleep disorders	• Insomnia: "prominent symptom" [4], p. 22, (29); "classic symptom" [8]; "principal feature" [9].<br>• "the most commonly reported syndrome includes […] insomnia, […] drowsiness" [8].<br>• "increased Hg° absorption was frequently associated with […] sleep disorder" [P].	Common	**Mod. & Sev.** (sleep disorders / insomnia)
					• "Tremor […] follows […] insomnia […]" [8].	Occurs earlier than tremor	
					• "[...] in all type [*differentiation in mild, medium, severe*] we found an problem with sleep" [I].	Occurs in moderate and severe case	
					• Insomnia occurs in "[…] workers exposed to elemental mercury vapour concentrations of the order of 0.1 mg/m^3"	Level of observation.	

					[8].		
70			*• Erethism / mercurial erythrism / erethismus / erethymus mercurialis [I] [P] [3] [5] [6] [7] [8] [9]<br>• neuropsychological changes / symptoms [I] [P]<br>• psychotic symptoms [6]*	*• erethism*	*• Erethism: „major clinical features" [5], p. 347; "main feature" [6], p. 619; "classic symptom" [8]; "principal feature" [9].<br>• Erethism: main symptom, most important [I].*	*Common*	Incl. of sub-ordinated symptoms
					• First erethism [I].<br>• "[…] the most pronounced [symptom] is […] erethism" [I].	*Early sign*	
					• "[…] tremor and erethism come together" [I].	*Occurs together with tremor*	
					• "At a urinary mercury excretion level of 100 µg per g creatinine, the probability of developing […] erethism […] is high" [9].<br>• "no evidence of the classical symptoms of […] erethism […] below a time-weighted occupational exposure to mercury in air of 100 µg/m^3" [9].	*Level of observation.*	
					• "Occupational exposure has resulted in erethism, with irritability, excitability, excessive shyness, and insomnia as the principal features of a broad-ranging functional disturbance. […] Long-term, low-level exposure has been found to be associated with less pronounced symptoms of erethism, characterized by fatigue, irritability, loss of memory, vivid dreams, and depression" [9].	*Severity is lower in long-term, low level exposures; Symptoms of erethism are different depending on the severity*	
					• Erethism: "Individual variation in exposed people is the rule […]" [8].	*Severity differs*	
71			• cortical hyperexcitability [P]<br>• disturbances [I]<br>• excitability [7] [8] [9]<br>• hyperirritability [I] [P]<br>• irritabile [I]<br>• irritability [P] [3] [4] [5] [6] [8] [9]<br>• mental hyperactivity [5]<br>• morbid irritability [5]<br>• restlessness [P]	• irritability	• Irritability: "prominent symptom" [4], p. 22; "principal features" [9]; "classic symptom" [8].<br>• "the most commonly reported syndrome includes […] irritability" [8].	Common	**Mod. & Sev.** (irritability / hyperirritability)
					• "[…] the irritability manifested more in middle and heavy [cases]" [I].<br>• Irritability: "may still persist" [6].	Not one of the early signs, but present in middle and heavy cases.	
72			• explosive loss of temper when criticized (as reaction of pathological fear of ridicule) [6]<br>• outbursts of temper [5]<br>• lack of self-control [8]	• lack of self-control	• "the most commonly reported syndrome includes […] lack of self-control" [8].	Common. No distinction in moderate and severe cases possible.	**Mod.**
73			• nervousness [3] [4] [8]		• Nervousness: "prominent symptom" [4], p. 22.	Common. No distinction in moderate and	**Mod. & Sev.**

						severe cases possible.	
					• Nervousness occurs "in workers exposed to elemental mercury vapour concentrations of the order of 0.1 mg/m^3" [8].	Level of observation.	
					• ""Tremor […] follows […] nervousness […]" [8].	Occurs earlier than tremor	
74			• sadness [I] [P]		/	Very few sources.	*[just mentioned in the DIWIntox meeting; not in the literature]*
75	**Renal effects**		Synonyms / summarizing symptom categories<br>• abnormal renal function [2]<br>• changes in renal function [4]<br>• damage to the kidney [2]<br>• effects on (the) kidney [6] [8] [9]<br>• impairment of kidney [I]<br>• kidney damage [6]<br>• kidney effects [9]<br>• proximal tubular changes / damage [3]<br>• renal changes [4]<br>• renal damage [4]<br>• renal effects [3] [4]<br>• renal toxicity [3]<br>• tubular damage [5]<br>• tubular dysfunction [I] [P] [4]<br>• tubular effects [9]		• Kidney: "major target organ" [3], p. 33; "notable target organ" [5], p. 347, "critical organ" [I].	Common	**Mod. & Sev.** (renal effects / abnormal renal function)
					• "Long-term exposure […] may lead to changes in renal function" [4], p. 30.	Result of long-term exposure	
					• "[…] clinically significant renal damage […] has not been reported at exposure levels normally encountered in the workplace" [4], p. 30.<br>• „Severe kidney damage sometimes associated with the nephrotic syndrome may also be present." [6], p. 619.	Not common: renal damage	
					• "[…] adverse effects in other organs […] occur at exposure levels higher than those affecting the […] kidneys" [4], p. 30.	Occurs in lower exposures than in other organs	
					• "Effects […] on the kidney had been reported only at doses higher than those associated with the onset of CNS signs and symptoms" [8] [9]. Since then several new studies have been carried out, and kidney effects have been seen at lower exposure levels" [9].<br>• „Slight renal involvement (proteinuria, albuminuria, enzymuria) may be detectable earlier than neurological involvement" [2].	Occurs earlier than neurological symptoms	
					• „The renal effects were mainly found in workers excreting more than 50 µg mercury/g creatinine" [4], p. 28.	Level of observation.	
					• „At higher levels (above 50 µg/g (micrograms per gram) abnormal renal function (as evidenced by N-acetyl-B-D-glucosaminidase (NAG), which is a sensitive indicator of damage to the kidneys) have been observed" [2].	Severe cases (abnormal renal function with enhanced NAG)	
76			• glomerular changes [3]<br>• glomerular dysfunction [I] [P] [9]<br>• glomerulosclerosis [3]	• glomerular dysfunction	/	No distinction in moderate and severe cases possible.	**Mod. & Sev.**

77		• nephrotic syndrome [5] [6] [9]<br>• albuminuria due to nephrotic syndrome [6]<br>• edema due to nephrotic syndrome [6]	• nephrotic syndrome	• „The nephrotic syndrome is an idiosyncratic reaction characterized by albuminuria and edema. Generally speaking such severe cases are rare and only found with chronic exposures, usually in the range of 500 µg Hg/m3 and higher […]" [6].<br>• Nephrotic syndrome" „less commonly" [9]; "can occur in severe cases" [5], p. 348.	Not common; severe cases. Level of observation.	Excl.
78		• urinary dysfunction [3]		/	Very few sources.	Excl.
79		Signs<br>• increases in urinary excretion of tubular antigens [3]		• "The main renal changes associated with exposure to mercury […] increased leakage of tubular antigens […]" [4], p. 28.	Common renal sign.<br>Very few sources.	Excl.
80		Signs<br>• changes in the specific gravity of the urine [3]		/	Very few sources.	Excl.
81		Signs<br>• decreased urinary excretion of some eicosanoids [4]<br>• decreased excretion of<br>- prostaglandin E2 and F2α [3]<br>- thromboxane B2 [3]	• decreased excretion of molecules	• "The main renal changes associated with exposure to mercury were […] biochemical alterations (decreased urinary excretion of some eicosanoids […])" [4], p. 28.	Common renal sign.<br>Very few sources.	Excl.
**82**		Signs<br>• decreased urinary excretion of some glycosaminoglycans [3] [4]		• The main renal changes associated with exposure to mercury were […] biochemical alterations (decreased urinary excretion of some […] glycosaminoglycans)" [4], p. 28.	Common renal sign.<br>Very few sources.	Excl.
**83**		Signs<br>• decreases in urinary pH [3]<br>• lowering of urinary pH [4]	• decreased urinary pH	• The main renal changes associated with exposure to mercury […] lowering of urinary pH" [4], p. 28.	Common renal sign.<br>Very few sources.	Excl.
**84**		Signs<br>• increased plasma concentrations of beta-galactosidase [9]		/	Very few sources.	Excl.
**85**		Signs<br>• increased plasma concentrations of beta-2-microglobulin [9]<br>• increased levels of certain lysosomal enzymes in plasma [9]	• increased plasma concentrati ons of proteins	• "Increased levels of certain lysosomal enzymes were found in plasma, and this effect was observed even in a group where the average urine mercury level was only 35 µg/litre" [9].	Level of observation.<br>Occurs in low exposures.<br>Level of observation.<br>Very few sources.	Excl.
**86**		• tubular cytotoxicity (increased leakage of tubular antigens and enzymes into urine) [4]	• enzymuria	• "The main renal changes associated with exposure to mercury were indicative of tubular cytotoxicity (increased leakage of tubular antigens and enzymes into urine" [4], p.	Common renal sign. No distinction in moderate and	**Mod. & Sev.**

		Signs<br>• increased urinary excretion of<br>- ß-/beta-galactosidase [4] [9]<br>- lysosomal enzymes in the urine [9]<br>• enzymuria [2]		28.	severe cases possible.	
				• „Slight renal involvement ([…] enzymuria) may be detectable earlier than neurological involvement“ [2].	Occurs earlier than neurological involvement	
				• „excretion of several proteins […] with urinary mercury levels in excess of 50 µg/g creatinine (ß-galactosidase, even among workers with urinary mercury >20 µg/g creatinine) […]" [4].<br>• „[…] an increased concentration of beta-galactosidase even in the group of workers with an average urine mercury concentration of about 20 µg/g creatinine" [9].	Level of observation.	
**87**		Signs<br>• increased urinary excretion of<br>- albumin [3] [4] [9]<br>- ß2-microglobulin [P] [4]<br>- high and low molecular weight urinary protein [P]<br>- proteins of low relative molecular mass [9]<br>- proteins with high relative molecular mass [9]<br>- retinol-binding proteins [9]<br>- several proteins [4]<br>- transferrin [4] [9]<br>- Tamm-Horsfall glycoprotein [3]<br>• increases in urinary protein [3]<br>• albuminuria [2] [3] [9]<br>• glomerular proteinuria [9]<br>• microalbuminuria [9]<br>• proteinuria [I] [P] [2] [3] [4] [5] [8] [9]<br>• tubular proteinuria [I]	• proteinuria	• "Proteinuria is the most common sign of the kidney effects due to tubular damage" [5], p. 348.	Common sign of the tubular damage.	**Mod. & Sev.** (proteinuria / high proteinuria)
				• "Occupational exposure to metallic mercury has long been associated with the development of proteinuria, both in workers with other evidence of mercury poisoning and in those without such evidence“ [9].	Early sign.	
				• "[…] a significant correlation between urinary mercury excretion and protein excretion have been demonstrated“ [9].<br>• "The urinary excretion of transferrin, albumin, and beta-galactosidase was significantly correlated with the urine concentration of mercury“ [9].<br>• "The urinary protein correlated with urinary mercury levels“ [8].	Severity rises with exposure.	
				• "[…] heavy albuminuria was reported to be accompanied by both proximal tubular damage and glomerulosclerosis." [3], p. 5.<br>• "[…] high level of proteinuria" [I].	Rising severity (high proteinuria).	
				• Proteinuria "[…] have been reported but only at doses higher than those associated with the onset of signs and symptoms from the central nervous system" [8].	Occurs later than CNS symptoms.	
				• „Slight renal involvement (proteinuria, albuminuria […]) may be detectable earlier than neurological involvement“ [2].	Occurs earlier than CNS symptoms.	
				• "At a urinary mercury excretion level of 100 µg per g creatinine, the probability of developing […] proteinuria is high" [9].	Level of observation	

**88**		Signs<br>• increased urinary excretion of<br>- N-acetyl-β-glucosaminidase (NAG) [2] [3] [4] [6] [9]	• increased urinary excretion of NAG	• "[…] Perhaps the most consistent finding is an increase in urinary excretion of N-acetyl-β-d-glucosaminidase (NAG)", […] where average urine levels are generally below 50 µg Hg/L. […] average urine levels covered the range of 6 to 115 µg Hg/L" [6], p. 619f.	Common.	**Mod. & Sev.** (Increased urinary excretion of NAG / rising urinary excretion of NAG)
				• „At higher levels (above 50 µg/g (micrograms per gram) abnormal renal function (as evidenced by N-acetyl-B-D-glucosaminidase (NAG), which is a sensitive indicator of damage to the kidneys) have been observed" [2].<br>• "[…] urinary *N*-acetyl-beta-glucosaminidase (NAG) enzyme levels increased with increasing urine mercury levels over the range of 100-250 µg/litre "[9].	Level of observation. Rising severity.	
**89**		Signs<br>• subclinical urea [I]	• subclinical urea level	/	Very few sources.	Excl. *[just mentioned in the DIWIntox meeting; not in the literature]*
**90**	**Respiratory effects**	• cough [7]		/	Very few sources.	Excl.
**91**		• damage to the lining of the lungs [3]		/	Very few sources.	Excl.
**92**		• dyspnea [7]		/	Very few sources.	Excl.

References

References of Supplementary File 2

Andersen, A., Ellingsen, D. G., Morland, T., & Kjuus, H. (1993). A neurological and neurophysiological study of chloralkali workers previously exposed to mercury vapour. *Acta Neurol Scand, 88*(6), 427-433.

ATSDR (1999). Toxicological Profile for Mercury. Atlanta: Agency for Toxic Substances and Disease Registry.

Barregard, L., Horvat, M., & Schutz, A. (1994). No indication of in vivo methylation of inorganic mercury in chloralkali workers. *Environ Res, 67*(2), 160-167, doi:10.1006/enrs.1994.1071.

DFG (2000). *MAK- und BAT-Werte-Liste*. Weinheim: Wiley VCH.

Ellingsen, D. G., Barregard, L., Gaarder, P. I., Hultberg, B., & Kjuus, H. (1993a). Assessment of renal dysfunction in workers previously exposed to mercury vapour at a chloralkali plant. *Br J Ind Med, 50*(10), 881-887.

Ellingsen, D. G., Morland, T., Andersen, A., & Kjuus, H. (1993b). Relation between exposure related indices and neurological and neurophysiological effects in workers previously exposed to mercury vapour. *Br J Ind Med, 50*(8), 736-744.

Falnoga, I., Kobal, A. B., Stibilj, V., & Horvat, M. (2002). Selenoprotein P in subjects exposed to mercury and other stress situations such as physical load or metal chelation treatment. *Biol Trace Elem Res, 89*(1), 25-33, doi:10.1385/bter:89:1:25.

Falnoga, I., Tusek-Znidaric, M., Horvat, M., & Stegnar, P. (2000). Mercury, selenium, and cadmium in human autopsy samples from Idrija residents and mercury mine workers. *Environ Res, 84*(3), 211-218, doi:10.1006/enrs.2000.4116.

Hribernik, I. (1950). [Our observations on occupational intoxication with mercury in Idrija (1946-1950), in Slovene]. *Arh Hig Rada Toksikol, 1*, 291-299.

Kobal, A. (1975). [Evaluation of the effectivenss of personal protective devices at working sites with high concentrations of mercury vapors]. *Zentralbl Arbeitsmed, 25*(12), 366-371.

Kobal, A. (1991). *[Occupational exposure to elemental mercury and its influence on mercury in blood, erythrocytes, plasma, exhaled breath and urine and catalase activity in erythrocytes, in Slovene]* Ljubljana University, Ljubljana.

Kobal, A. (1994). [Mercury in Idria - an overview of the past and present from the aspect of occupational medicine in German]. *Zbl Arbeitsmed, 44*, 200-210.

Kobal, A., & Bohinjec, M. (1982). [Beta microglobulinuria in workers exposed to inorganic mercury at the Idrija mercury mine, in Slovene]. *Zdrav Vestnik, 51*, 559-602.

Kobal, A., Horvat, M., Hieng, N., & Stegnar, P. Catalase activity in the blood of workers exposed to Elemental Mercury. In *Mercury as Global polutant – 5th Inernational coference May 23-28, 1999, Rio de Janeiro, Brasil, 1999*

Kobal, A., Nanut, E., Horvat, M., Stegnar, P., & Sabadin, A. (1999). Evaluation of internal Doses of Mercury at Intermitent Exposure to Elemental Mercury at the mine Idrija. In R. Ebinghaus, R. R. Turner, L. D. De Lacerda, O. Vasiliev, & W. Salomons (Eds.), *Mercury Contaminated Sites* (pp. 271-279). Berlin, Heidelberg: Springer.

Kobal, A. B., Flisar, Z., Miklavcic, V., Dizdarevic, T., & Sesek-Briski, A. (2000). Renal function in miners intermittently exposed to elemental mercury vapour. *Arh Hig Rada Toksikol, 51*(4), 369-380.

Kobal, A. B., Horvat, M., Prezelj, M., Briski, A. S., Krsnik, M., Dizdarevic, T., et al. (2004). The impact of long-term past exposure to elemental mercury on antioxidative capacity and lipid peroxidation in mercury miners. *J Trace Elem Med Biol, 17*(4), 261-274, doi:10.1016/s0946-672x(04)80028-2.

Kobal, A. B., & Kobal Grum, D. (2010). Scopoli's work in the field of mercurialism in light of today's knowledge: past and present perspectives. *Am J Ind Med, 53*(5), 535-547, doi:10.1002/ajim.20798 [doi].

Kobal, A. B., & Kobal Grum, D. (2012). Elemental mercury Exposure and Sleep Disorder. In C. Idzikowski (Ed.), *Sleep Disorders* (pp. 47-64). Rijeka: In Tech

Kobal, A. B., Prezelj, M., Horvat, M., Krsnik, M., Gibicar, D., & Osredkar, J. (2008). Glutathione level after long-term occupational elemental mercury exposure. *Environ Res, 107*(1), 115-123, doi:10.1016/j.envres.2007.07.001.

Kobal Grum, D., Kobal, A. B., Arneric, N., Horvat, M., Zenko, B., Dzeroski, S., et al. (2006). Personality traits in miners with past occupational elemental mercury exposure. *Environ Health Perspect, 114*(2), 290-296.

Kosta, L., Byrne, A. R., & Zelenko, V. (1975). Correlation between selenium and mercury in man following exposure to inorganic mercury. *Nature, 254*(5497), 238-239.

Kosta, L., Zelenko, V., Stegnar, P., Ravnik, V., Dermelj, M., & Byrne, A. (1972). Fate and significance of mercury residues in an agricultural ecosystem. *IAEA-PL, 469*(5), 47-49.

Letz, R., Gerr, F., Cragle, D., Green, R. C., Watkins, J., & Fidler, A. T. (2000). Residual neurologic deficits 30 years after occupational exposure to elemental mercury. *Neurotoxicology, 21*(4), 459-474.

Lund, B. O., Miller, D. M., & Woods, J. S. (1993). Studies on Hg(II)-induced H2O2 formation and oxidative stress in vivo and in vitro in rat kidney mitochondria. *Biochem Pharmacol, 45*(10), 2017-2024.

Mathiesen, T., Ellingsen, D. G., & Kjuus, H. (1999). Neuropsychological effects associated with exposure to mercury vapor among former chloralkali workers. *Scand J Work Environ Health, 25*(4), 342-350.

Roels, H., Gennart, J. P., Lauwerys, R., Buchet, J. P., Malchaire, J., & Bernard, A. (1985). Surveillance of workers exposed to mercury vapour:validation of a previously proposed biological threshold limit value for mercury concentration in urine. *Am J Ind Med, 7*(1), 45-71.

Schaller, K., Breininger, M., & R, S. (1983). Der Quecksilberspiegel für Blut und Urin bei Normalpersonen. *Ärztliches Laboratorium, 29*, 325-334.

Scopoli, J. A. (1761): De Hydrargyro Idriensi Tentamina.

WHO (1976). Mercury. Environmental Health Criteria 1. Geneva: World Health Organization, International Programme on Chemical Safety.

WHO (1980). Recommended hearth-based Limits in occupationat exposure to heavy metats. Report of a WHO Study Group. Geneva: World Health Organization.

WHO (1991). Inorganic Mercury. Environmental Health Criteria 118. Geneva: World Health Organization.

WHO (2003). Elemental Mercury and Inorganic Mercury Compounds: Human Health Aspects. Concise International Chemical Assessment Document 50. Geneva: World Health Organization.

References of Supplementary File 5

[I] Information from the interview during the DiWIntox expert meeting, December 2012, Ljubljana, Slovenia.

[P] Information from the presentation of Alfred Kobal (with references to Scopoli, Paracelsus and others; see Online Resource 2 for a list of references) during the DiWIntox meeting, December 2012, Ljubljana, Slovenia.

1. Knol AB, Slottje P, van der Sluijs JP, Lebret E: The use of expert elicitation in environmental health impact assessment: a seven step procedure. *Environ Health* 2010, 9:19.
2. Nordberg G: 63. Metals: Chemical Properties and Toxicity - Mercury. In: *ILO Encyclopaedia of Occupational Health & Safety.* Edited by Nordberg CEG, vol. Fourth Edition; 1998.
3. ATSDR: Toxicological Profile for Mercury. In. Edited by U.S. Department of Health and Human Services, Public Health Service, Agency for Toxic Substances and Disease Registry; 1999.
4. WHO: Elemental Mercury and Inorganic Mercury Compounds: Human Health Aspects. In: *Concise International Chemical Assessment Document 50.* Geneva; 2003.
5. Park JD, Zheng W: Human exposure and health effects of inorganic and elemental mercury. *Journal of preventive medicine and public health = Yebang Uihakhoe chi* 2012, 45(6):344-352.
6. Clarkson TW, Magos L: The toxicology of mercury and its chemical compounds. *Critical reviews in toxicology* 2006, 36(8):609-662.
7. Guzzi G, La Porta CA: Molecular mechanisms triggered by mercury. *Toxicology* 2008, 244(1):1-12.
8. WHO: Mercury. Environmental Health Criteria 1. In.: WHO IPCS INCHEM (International Programme on Chemical Safety); 1976.
9. WHO: Inorganic Mercury. Environmental Health Criteria 118. In.: WHO IPCS INCHEM (International Programme on Chemical Safety); 1991.
